# Evaluating the transcriptional landscape and cell-cell communication networks in chronically irradiated parotid glands

**DOI:** 10.1016/j.isci.2023.106660

**Published:** 2023-04-11

**Authors:** Brenna A. Rheinheimer, Mary C. Pasquale, NIDCD/NIDCR Genomics and Computational Biology Core, Kirsten H. Limesand, Matthew P. Hoffman, Alejandro M. Chibly

**Affiliations:** 1 Matrix and Morphogenesis Section, National Institute of Dental and Craniofacial Research, National Institutes of Health, Bethesda, MD 20892, USA; 2 Nutritional Sciences Department, University of Arizona, Tucson, AZ 85721, USA; 3 Genomics and Computational Biology Core, National Institute on Deafness and Other Communication Disorders, 35A Convent Drive, Room 1F-103, Bethesda, MD 20892, USA

**Keywords:** Cell biology, Immunology, Transcriptomics

## Abstract

Understanding the transcriptional landscape that results in chronic salivary hypofunction after irradiation will help identify injury mechanisms and develop regenerative therapies. We present scRNA-seq analysis from control and irradiated murine parotid glands collected 10 months after irradiation. We identify a population of secretory cells defined by specific expression of *Etv1*, which may be an acinar cell precursor. Acinar and *Etv1*+ secretory express *Ntrk2* and *Erbb3,* respectively while the ligands for these receptors are expressed in myoepithelial and stromal cells. Furthermore, our data suggests that secretory cells and CD4^+^CD8^+^T-cells are the most transcriptionally affected during chronic injury with radiation, suggesting active immune involvement. Lastly, evaluation of cell-cell communication networks predicts that neurotrophin, neuregulin, ECM, and immune signaling are dysregulated after irradiation, and thus may play a role in the lack of repair. This resource will be helpful to understand cell-specific pathways that may be targeted to repair chronic damage in irradiated glands.

## Highlights

• *Etv1* defines a discrete cluster of secretory cells that may be acinar precursors
• Ntrk2 and Erbb3 are highly specific secretory cell receptors
• CD4^+^CD8^+^T-cells and acinar cells have the greatest transcriptional changes after IR
• Ntrk2, Erbb3, ECM, and immune signaling are altered in secretory cells after IR

## Graphical abstract

**Figure:**
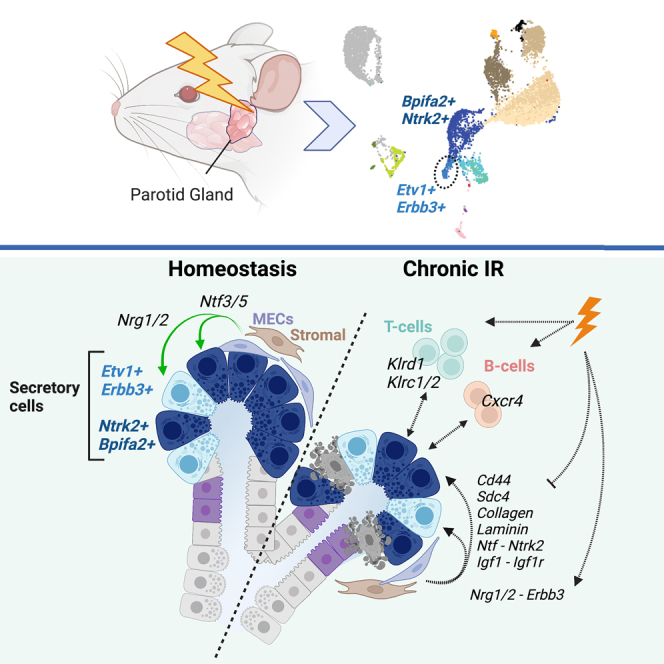

## Introduction

Of the three major pairs of salivary glands (SGs): the parotid (PG), submandibular (SMG), and sublingual (SLG), the human PG is the largest and produces the largest volume of saliva, particularly in response to gustatory simulation. In mice, the PG is smaller than the SMG but also contributes to the majority of stimulated saliva.^1^ In addition, the PG is thought to be the most sensitive to irradiation (IR) damage during treatment of head and neck cancer with radiotherapy,^2^^,^^3^ which results in permanent salivary hypofunction. In terms of understanding salivary gland biology, most studies have focused on the SMG both in the context of development and response to injury; however, each gland has unique functions and transcriptional profile.^4^ Here we set out to investigate the effects of irradiation damage to PGs in mice using single cell (sc)RNA-seq.

The PG primarily comprises serous acinar cells which produce large volumes of watery serous saliva that is transported through the ductal system into the oral cavity to aid in digestion and protection of mucosal surfaces. Despite advances in radiotherapy, it is estimated that ∼40% of head and neck cancer patients suffer from the chronic consequences of salivary gland damage months to years after the completion of radiotherapy, even with newer modalities such as intensity-modulated radiation treatment (IMRT) that reduces exposure to non-tumor tissues.^5^^,^^6^^,^^7^^,^^8^^,^^9^ Animal studies show that the acute effects of radiotherapy in the PG occur in the days and weeks following initial treatment and are likely a result of high levels of acinar cell death,^10^ DNA damage,^11^ dysregulated calcium signaling and ROS generation,^12^ inflammatory responses,^13^ and alterations to the nerves and vasculature, whereas the chronic effects arise months to years after initial treatment (Reviewed in Jasmer et al.^14^). Chronic loss of function is often attributed to fibrosis and the inability of acinar regeneration to occur, and preclinical studies suggest that persistent acinar cell proliferation, vascular damage, and parenchymal cell loss may be contributing factors.^15^^,^^16^^,^^17^^,^^18^ In a similar manner, patients with Sjogren’s syndrome, an autoimmune disease that damages the acinar cells of salivary and lacrimal glands, life-long consequences include dental caries, reduced taste and smell, malnutrition, mucositis, and increased risk for oral infections leading to a significant decrease in quality of life.^19^ Therefore, translational frameworks to understand chronic glandular dysfunction following IR therapy along with the development of regenerative therapies remains an unmet need.

The development of scRNA-seq has made it possible to identify previously uncharacterized cell types within a tissue and to uncover and gene regulatory networks and mechanisms regulating cell-cell communication and specific cell states.^20^^,^^21^^,^^22^^,^^23^ To date, there have been scRNA-seq studies performed for virtually all major tissues, including atlas-level scRNA-seq datasets such as the Tabula Muris^24^ or the Tabula Sapiens^25^ which integrate data from multiple organs in mouse and human, respectively. There are also numerous scRNA-seq studies on disease-specific models, which are important to understand the cellular mechanisms involved that could be targeted for repair or regeneration. In SGs, scRNA-seq studies have focused on understanding homeostasis and development,^26^^,^^27^^,^^28^^,^^29^^,^^30^ as well as particular disease states, such as cancer,^31^ Sjogren syndrome,^32^^,^^33^ and COVID-19 infection.^27^

In this study, we use scRNA-seq to characterize the adult mouse PG and compare the transcriptional landscape 10 months after IR damage to explore chronic dysfunction after irradiation. Owing to the complex heterogeneity of the SGs, distinguishing cell-type compositional differences and their specific and direct contribution to the loss of saliva following radiation therapy is complex, and single-cell transcriptomics will begin to resolve this issue.

This dataset allows for discovery and exploratory research into the mechanisms and cellular processes driving PG dysfunction after IR in a model of fractionated IR with limited acinar cell loss. Our work has been validated by immunofluorescence staining to confirm the presence of selected markers in specific cell populations, confirming the potential to reveal meaningful biological insights. It is noteworthy that scRNA-seq of *in vivo* models of chronic IR injury has only been performed in liver,^34^ lung,^35^ and skin,^36^ and data is only publicly available for lung and skin. Thus, our study will also be an essential resource to better understand cell-specific responses to IR in general.

## Results

### Generation of a single-cell resource of healthy and irradiated mouse parotid gland

**Figure 1:**
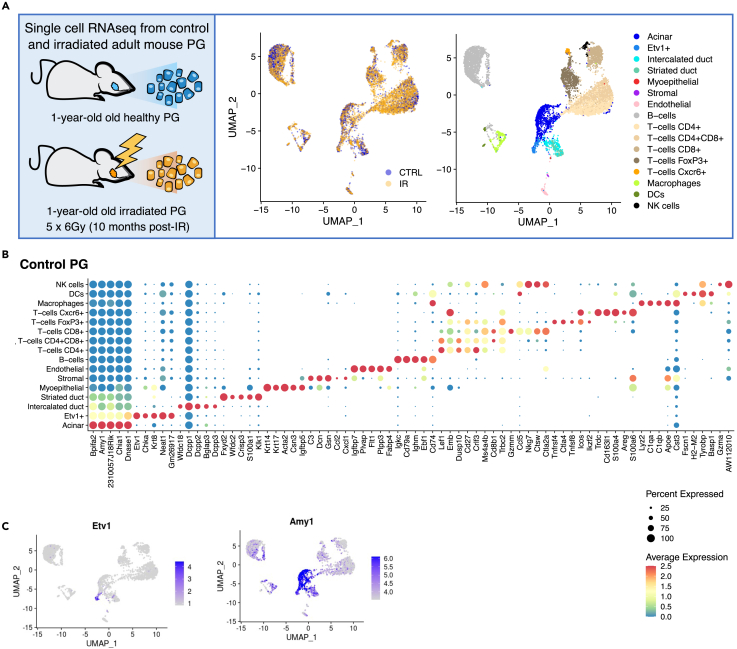
scRNA-seq analysis of control and irradiated PG (A) Single cell suspensions from 1-year-old control and irradiated PG from 2C3H female mice were used to build scRNA-seq libraries. Representative UMAP plots are colored by treatment group or cell type. Clusters were annotated based on the expression of known markers. (B) Balloon Plot with top 5 DEGs per cluster sorted by fold change. Statistical analysis was performed with SEURAT, which uses a non-parametric Wilcoxon rank-sum test for differential expression. Color is relative to scaled gene expression and size of the dot represents the percentage of cells expressing the gene. (C) Representative UMAP plots showing expression of Etv1 and Amy1.

Using the 10X Genomics platform, we generated 2 individual scRNA-seq libraries of healthy and IR mouse PG collected 10-month after irradiation (Figure 1A). Mice received 6 Gy IR/day to the head and neck region on five consecutive days, for a total dose of 30 Gy. This mouse model of IR damage to SGs results in chronic loss of saliva with partial loss of epithelial cells.^37^ Control and IR PG samples were bioinformatically integrated with SEURAT v4 and clustered following SEURAT’s standard workflow.^38^^,^^39^ The optimal resolution for clustering was determined using clustree package^40^ and the resulting 17 cell clusters were annotated based on their gene expression profile (Figures 1B, S1A, and S1B) and a previously generated atlas of SMG development which provided cell type specific markers.^28^ Stromal and myoepithelial cells (MECs) clustered together with endothelial cells likely because of the low number of cells recovered for these populations. Thus, they were manually annotated based on expression of a combination of stromal (*Col1a12* and *Vim*) and myoepithelial (*Krt14* and *Acta2*) markers which were highly specific (Figures S1C and S1D). We did not identify discrete clusters of basal duct cells (*Krt14+Krt5+*) or peripheral nerves presumably because of limitations in the dissociation technique, which has been previously reported for adult SG tissue dissociation.^28^

The identified populations included acinar cells (*Amy1+*), intercalated duct (*Dcpp1-3+*), striated duct (*Fxyd2+, Klk1+*), MECs (*Acta2+Krt14+*), stromal (*Col1a1+Vim+*), endothelial (*Pecam1+*), and 9 distinct immune populations including B-cells (*Cd79a+*and Immunoglobulin genes), five subtypes of T-cells (*CD4+; CD8+; CD4+CD8+; FoxP3+; Cxcr6+*), macrophages (*Adgre1+*), dendritic cells (*S100a8/9+*), and natural killer cells (*Gzma+Nkg7+*). We also identified a previously uncharacterized epithelial population defined by high expression of *Etv1* and *Krt8* and moderate expression of *Amy1* (Figures 1B, 1C, and S1B).

### Etv1 expression delineates a secretory sub-population in acinar and duct compartments

The two most striking observations from our initial clustering analysis are the identification of an *Etv1*+ epithelial population and the prominence of multiple resident immune cell types after IR. *Etv1* has been described as one of the top transcription factors expressed in the salivary glands.^41^ To characterize this *Etv1+*cluster, and to generate gene expression profiles of individual cell populations in healthy adult parotid glands, we performed differential expression analysis with SEURAT in the annotated control sample (Figure 1C). Genes enriched in a given cluster are herein referred to as cell-defining genes and were sometimes expressed elsewhere at lower levels. The complete gene list is included in Table S1.

**Figure 2:**
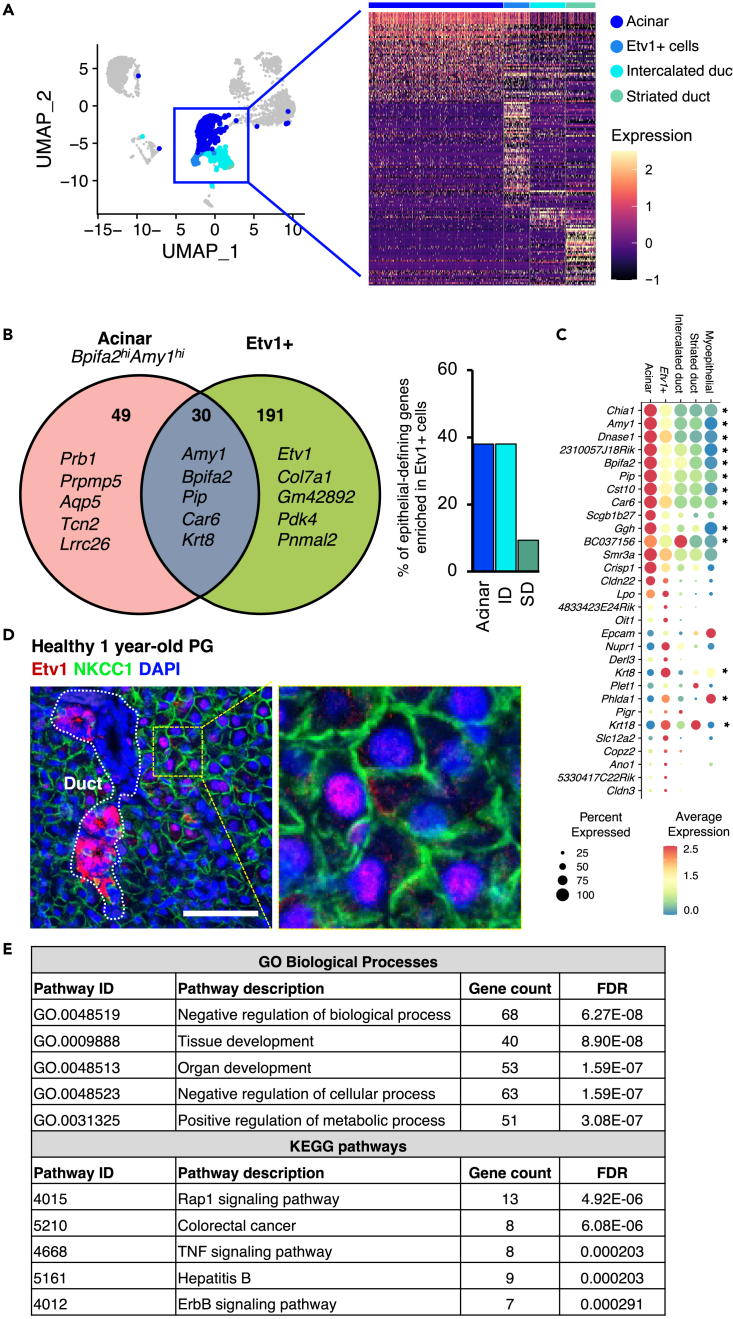
Characterization of acinar and Etv1+ cells (A) UMAP plot highlighting acinar, Etv1+, and duct populations with a representative heatmap of their gene expression profiles. Color scale bar represents scaled gene expression. (B) Venn diagram of cell-defining genes in acinar and Etv1+ clusters showing the number of unique and overlapping cell-defining genes. Representative genes from each group are shown. The bar graph shows the percentage of overlap between cell-defining genes in acinar and duct populations with Etv1+ cells. (C) Balloon plot showing expression of the 30 genes overlapping between acinar and Etv1+ cells. Genes marked with an asterisk are differentially expressed between Etv1+ and acinar cells (p<0.05, Wilcoxon rank-sum test (SEURAT)). Color is relative to scaled gene expression and size of the dot represents the percentage of cells within a cluster expressing the gene. (D) Immunofluorescence staining of PG from 1-year-old C3H mice stained for Etv1 (Red), NKCC1 (green) and DAPI (blue). The area delineated by the yellow dotted line is magnified to the right for visualization. Scale bar = 50um. (E) Results from STITCH analysis showing top biological processes and KEGG pathways associated with defining-genes from Etv1+ cells.

The expression of Amy1 in *Etv1+*cells suggested an acinar-like phenotype. When comparing the gene expression profile of major epithelial populations, 38% of acinar-defining genes (30 of 79) were enriched in *Etv1*+ cells (Figures 2A and 2B). Both cell types expressed serous secretory markers such as amylase (*Amy1*), parotid secretory protein (*Bpifa2*), prolactin induced protein (*Pip*), and carbonic anhydrase 6 (*Car6*), but their expression was significantly higher in acinar cells, while*Etv1+*cells had higher expression of *Krt8, Krt18,* and *Phlda1* (Figure 2C). When compared to duct populations, *Etv1+*cells expressed 38% (19 genes) of intercalated duct (ID)-defining genes (Figure S2A) and only 9.3% of striated duct (SD)-defining genes (Figures 2B and S2B), suggesting that *Etv1*+ cells are transcriptionally similar to both acinar and ID populations. Accordingly, Etv1 protein was detected by immunofluorescence in a subset of duct and acinar cells. Duct cells showed strong nuclear and cytoplasmic Etv1+ signal while it was predominantly nuclear in NKCC1+ acinar cells (Figure 2D).

Next, we performed functional analysis of all acinar and *Etv1*+ cell-defining genes using STITCH (search tool for interactions of chemicals, http://stitch.embl.de/), which integrates information about interactions from metabolic and KEGG pathways, crystal structures, binding experiments, and drug-target relationships.^42^ As expected, KEGG pathway analysis on acinar genes showed salivary secretion as one of the top pathways (Figure S2D). In contrast, in *Etv1*+ cells the top functions and pathways were associated with organ development and activation of Rap1, TNF, and ErbB signaling pathways (Figures 2E and S2C), suggesting that the *Etv1+*population has distinct functions despite their transcriptional similarities to acinar cells.

### Acinar and Etv1+ cells communicate with MECs and stromal cells via Erbb3 and Ntrk2

Given that cellular functions are often initiated by ligand-receptor interactions that trigger signaling cascades, we next used two bioinformatic approaches to predict putative cell-cell interactions: First, we used differential expression analysis for each cluster and cross-referenced the resulting cell-defining genes with a previously published database of curated ligand-receptor pairs.^43^ For reproducibility of this approach, we used R-scripted code which is available as supplemental information. In this database, a ligand is defined as any molecule that interacts with known receptors and thus intracellular components such as *Hras* are included. As a complementary approach, we used CellChat, which infers ligand-receptor pairs and associated pathways based on a manually-curated list of literature-supported interactions grouped into 229 signaling pathways.^44^ Both approaches were consistent and showed that acinar and duct cells had the lowest number of enriched ligand and receptor genes compared to all other cell types while MECs had the highest number across epithelial populations (Figures S3A–S3C).

**Figure 3:**
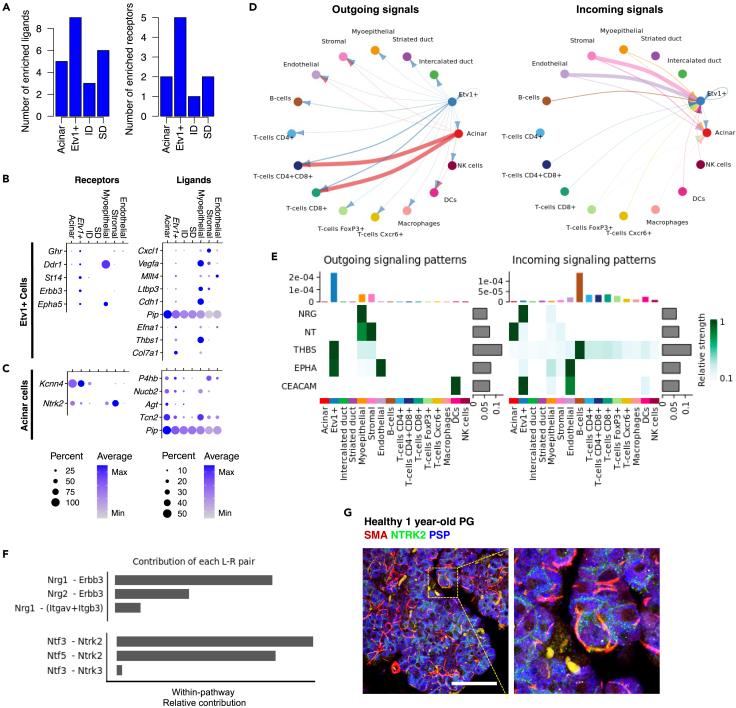
Ligand-receptor analysis of Etv1+ and acinar cells (A) Bar graphs with number of identified ligands and receptors among cell-defining genes from epithelial populations. (B) Balloon plots of expression of ligands and receptors enriched in Etv1+ cells. (C) Balloon plots of expression of ligands and receptors enriched in acinar cells. (D) Chord plots summarizing putative ligand-receptor interactions with Acinar and Etv1+ cells, as predicted by CellChat. The arrows point to the cell expressing the receptors and are colored based on the source of the ligand. The thickness of the arrow is relative to the number of putative pairs identified. (E) Heatmap shows the relative importance of each cell group based on CellChat-computed network centrality measures of NRG, NT, THBS, EPHA, and CEACAM signaling networks. (F) Relative contribution of each ligand-receptor pair to the overall communication network of NRG and NT signaling pathways, which is the ratio of the total communication probability of the inferred network of each ligand-receptor pair to that of the signaling pathway. (G) Immunofluorescence staining for Smooth muscle actin (SMA, Red), NTRK2 (green) and Parotid Secretory Protein (PSP, blue). The area delineated by the yellow dotted line is magnified to the right for visualization. Scale bar = 50um.

Differential expression analysis identified 9 ligand and 5 receptor genes among the *Etv1*+ cell-defining genes, as well as 5 ligands and 2 receptors in acinar cells (Figure 3A). The identified receptor genes enriched in *Etv1*+ cells included *Ghr*, *Dddr1*, *St14*, *Erbb3,* and *Epha5*, which were highly specific to this population (Figure 3B, left panel). On the other hand, the ligands found in *Etv1*+ cells were also enriched in other cell types, with the exception of *Col7a1,*which was highly specific (Figure 3B, right panel). A distinct set of ligands and receptors were enriched in acinar cells, including the receptor genes *Ntrk2* and *Kcnn4*, and the ligands *P4hb, Nucb2, Agt, Tcn2,* and *Pip.* All of the resulting putative interactions from our differential gene expression analysis are shown in Table S2. Interactions between acinar and *Etv1*+ cells with all other cell types are summarized as chord plots in Figures S3D and S3E. All interactions predicted by CellChat are available in Table S3. Based simply on the total number of possible pairs (without accounting for the level of expression of individual genes), the strongest outgoing interactions from *Etv1*+ cell ligands were predicted to occur with receptors in endothelial cells, whereas *Etv1*+ cell receptors corresponded to ligands from myoepithelial and stromal cells. In contrast, the corresponding pairs for acinar cell ligands were expressed primarily in T-cells (Figure S3D). These findings were largely corroborated by CellChat (Figure 3D), which does take into account the level of gene expression as well as the proportion of cells expressing a given ligand-receptor pair in a cluster.

CellChat analysis determined that Etv1+ cells were a source of ligands for Thrombospondin (THBS) and EphrinA (EPHA) signaling pathways, and had receptors for Carcinoembryonic antigen cell adhesion molecule (CEACAM) and Neuregulin (NRG) ligands, whereas acinar cells were receptive to Neurotrophin (NT) signaling (Figure 3E). These results revealed notable interactions between myoepithelial and Etv1+ cells via the *Erbb3* receptor and two of its ligands, Neuregulin1 (*Nrg1*) and *Nrg2*, and between myoepithelial and acinar cells via the neurotrophin receptor *Ntrk2* and one of its ligands, Neurotrophin 5 (*Ntf5*) (Figures 3E, 3F, S3D, and S3E). *Ntrk2* was also expressed in *Etv1*+, myoepithelial and stromal cells in our scRNA-seq data but immunofluorescence staining confirmed enrichment of the receptor in acinar cells of mouse parotid gland (Figure 3F). The cellular functions of *Ntrk2* in acinar cells are currently unknown and thus further mechanistic studies are warranted. Note that MECs, and to some extent, stromal cells, were recovered at a relatively low frequency, and thus CellChat’s results involving these populations should be interpreted with caution, although they are consistent with our previous observations that MECs and stromal cells are major sources of neurotrophins and other ligands in mouse SMG.^28^^,^^45^

### CD8^+^CD4^+^T-cells and acinar cells have the greatest transcriptional response to IR

**Figure 4:**
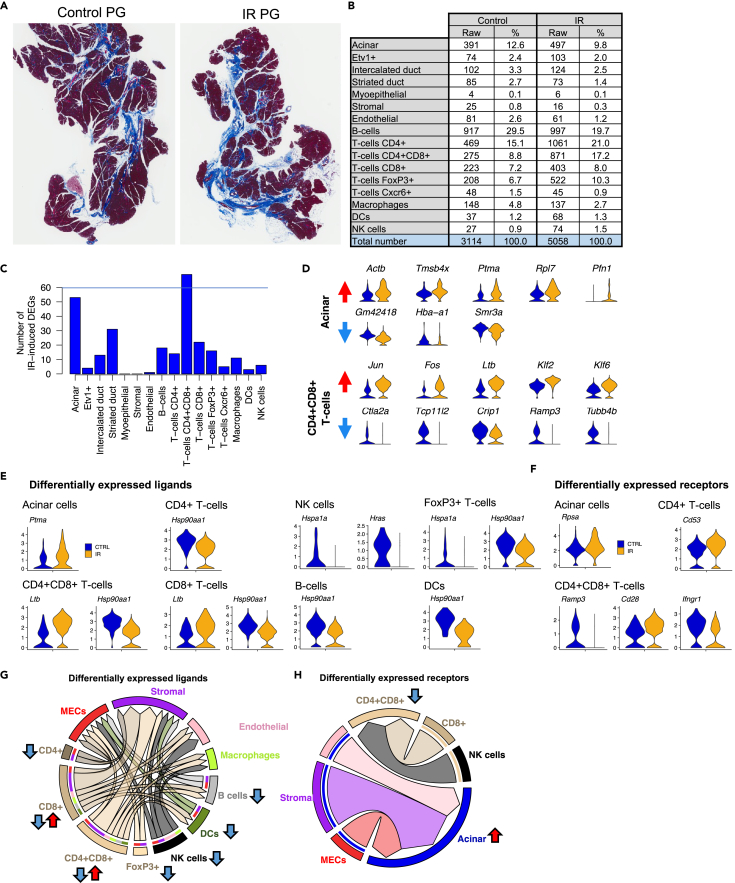
Cell-specific IR-induced DEGs (A) Masson’s trichrome staining of control and IR-PG collected 10 month after IR. Areas in blue represent fibrotic/collagenous tissue. (B) Cell numbers and proportions in scRNA-seq datasets from control and irradiated PG. (C) Bar graph showing number of DEGs after IR in individual cell populations. DE analysis was performed with SEURAT’s default Wilcoxon test (p<0.05). (D) Violin plots of top 5 (if present) up and downregulated genes in acinar and CD4^+^CD8^+^T-cells. Red and blue arrows denote upregulated and downregulated genes, respectively. (E and F) Violin plots of differentially expressed ligands and receptors from SEURAT’s DE analysis. (G and H) Chord plots of ligand-receptor interactions with IR-induced DE receptors and ligands.

The model of SG IR used in this study is based on a fractionated dosing schedule of 6Gy x 5 consecutive days, which leads to significant loss of saliva^37^ but it does not result in extensive loss of acinar cells and development of fibrosis (Figure 4A) as reported by Ferreira et al. While this model shows a milder phenotype compared to alternative models using a single 15Gy dose in distinct mouse strains,^12^^,^^46^^,^^47^^,^^48^ it has been previously used to demonstrate the therapeutic potential of adenovirus-based Neurturin gene transfer in the SMG to prevent the loss of saliva caused by IR. Given that we did not perform multiple technical replicates of each treatment, potential changes in cell proportions are reported as trends. In general, B cells and T cells were the most affected (Figures 4A and 4B). We observed a 33% relative decrease in the proportion of B cells, a 39% increase in CD4^+^T cells, and a 195% increase in CD4^+^CD8^+^T cells. A 22% decrease in the proportion of acinar cells was also noted.

Differential expression analysis with SEURAT was performed between control and irradiated cell types. The complete list of differentially expressed genes (DEGs) is shown in Table S4. CD4^+^CD8^+^T-cells had the highest number of dysregulated genes (∼70) after IR across all identified cell populations, followed by acinar cells (Figure 4C). We did not detect DEGs in MEC and stromal populations after IR, and only 1 gene was differentially expressed in IR endothelial cells. The lack of DEGs in MECs is likely explained because of the low number of MECs analyzed (Figure 4B). Stromal and endothelial cells also did not show significant changes in gene expression, but they were well-represented in our dataset; thus, cell numbers alone are not likely to account for the lack of DEGs after IR in these populations. Instead, the lack of DEGs may reflect the fact that this model of IR damage is not highly fibrotic (Figure 4A). Alternatively, it is possible that endothelial and stromal populations may have recovered a year after IR damage.

The top upregulated genes in acinar cells after IR included *Actb, Tmsb4x*, and *Pfn1*which are involved in actin polymerization (Figure 4D). The genes *Gm42418, Hba-a1*, and *Smr3a* were the only downregulated genes in acinar cells and they were also downregulated in most other cell types (Figure S4A and Table S4), suggesting a global response to IR rather than an acinar-specific one. In CD4^+^CD8^+^T-cells, the top upregulated genes after IR were *Jun, Fos, Ltb*, *Klf2*, and *Klf6*, and the most downregulated genes were *Ctla2a, Tcp11L2, Crip1, Ramp3,* and *Tubb4b* (Figure 4D). In general, DEGs in acinar cells were associated with regulation of transepithelial transport, electron transport, apoptosis, and translation processes according to gene ontology analysis via The Gene Ontology Consortium,^49^ while DEGs in CD4^+^CD8^+^T-cells were associated with V(D)J recombination, lymphocyte differentiation, apoptosis, axonogenesis, and ERK signaling pathway (Figure S4B).

When we cross-referenced the IR DEGs against the database of ligand-receptor pairs from Ramilowsky et al., only a handful of ligands and receptors were represented (Figures 4E and 4F), and only a few of these had a corresponding pair (Figures 4G, 4H, and Table S5). Most differentially expressed pairs were found between immune populations, MECs, stromal, and endothelial cells. In acinar and CD4^+^CD8^+^T-cells, which were the most transcriptionally affected, we identified 5 ligands (*Ptma, Hsp90aa1, Ltb, Hspa1a,* and *Hras*) and 5 receptor genes (*Rpsa*, *Cd53, Ramp3, Cd28*, and *Ifngr1*) differentially expressed after IR (Figures 4E and 4F). However, these DEGs were expressed across multiple clusters and were not defining for any individual population. For instance, *Hsp90aa1* was downregulated in all immune populations except NK cells and macrophages, and both *Hspa1a* and *Hras* were downregulated in NK cells (Figure 4E). Similarly, *Rpsa* was upregulated in acinar cells while*Ifngr1* was downregulated in CD4^+^CD8^+^T-cells after IR (Figure 4F). Putative pairs were found for *Rpsa* (Ribosomal protein SA (*Rpsa*), also known as Laminin receptor 1)*, Ifngr1* (Interferon Gamma Receptor 1)*, Hsp90aa1* (Heat shock protein 90 Alpha Family Class A Member 1)*, Ltb* (Lymphotoxin Beta), and *Hras* oncogene (Table S5).

This analysis suggested multiple signaling alterations including interactions with acinar cells via *Lamb2-Rpsa* and between NK and CD8^+^ cells with CD4^+^CD8^+^T-cells via *Ifng-Ifngr1* (Table S5, Figures 4G and 4H). Paracrine signaling via *Hsp90aa1* from immune cells to *Egfr* expressed in myoepithelial, stromal, and endothelial cells was potentially reduced, while*Ltb* interaction with *Tnfrsf1a* and *Cd40* expressed by macrophages, endothelial cells, dendritic cells, and B-cells was potentially increased.

### Neurotrophin, neuregulin, ECM, and immune signaling are the main altered pathways in Acinar and Etv1+ cells after IR

**Figure 5:**
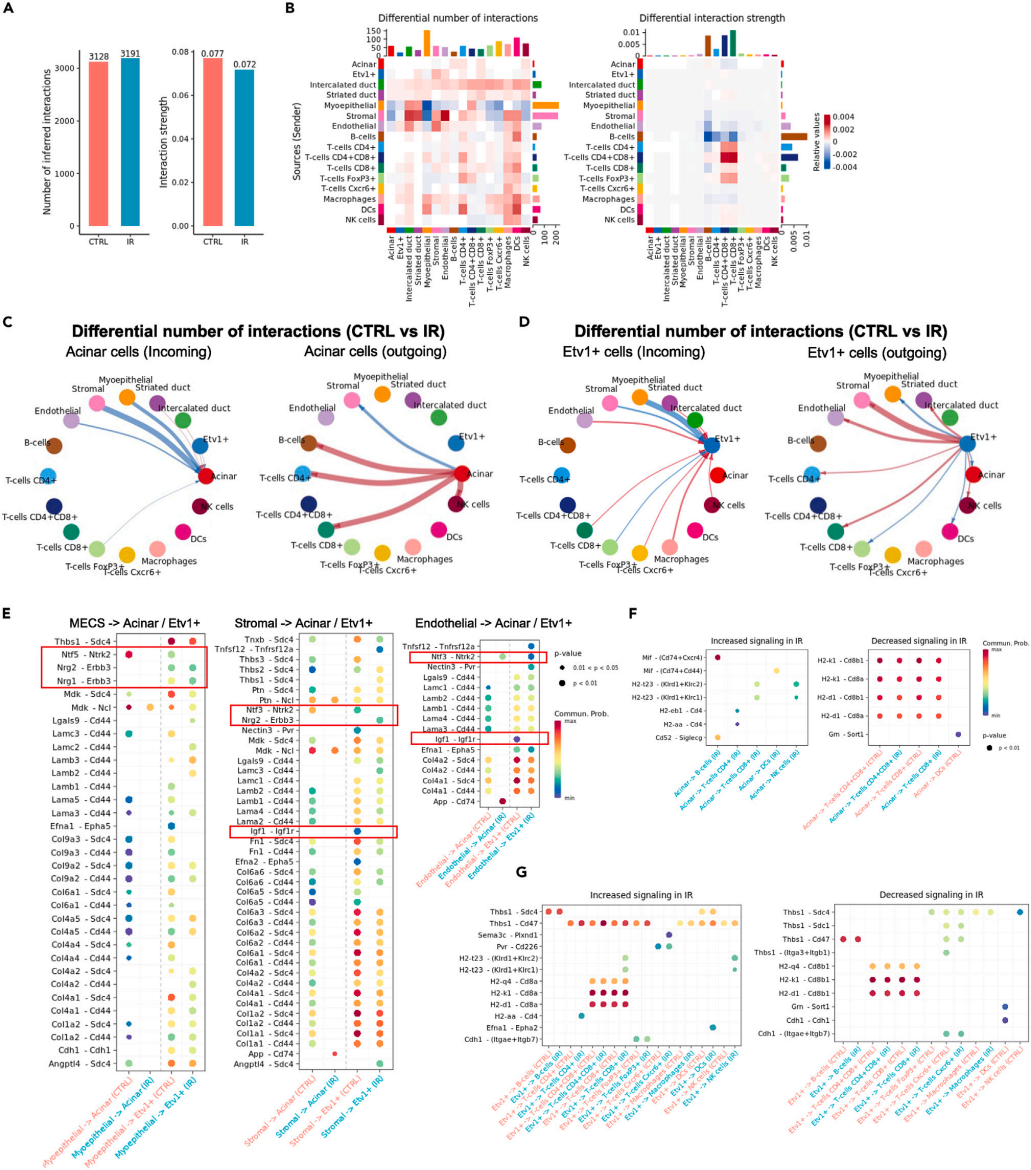
Dysregulated ligand-receptor pairs after IR (A) Number of ligand-receptor pairs and interaction strength in control and IR-PG determined by CellChat. (B) Differential number of possible interactions between acinar and Etv1+ cells with all other populations in IR-PG compared to control. Red (positive values) and blue (negative values) in the color bar indicate higher number of predicted interactions in IR-PG and controls, respectively. (C and D) Differential ligand-receptor interactions in Acinar and Etv1+ cells between IR-PG and control, as predicted by CellChat. The arrows point to the cell expressing the receptors. Red arrows indicate increased number of interactions and blue arrows represent a decrease. The thickness of the arrow is relative to the change in number of interactions between control and IR-PG. (E) Comparison of the significant ligand-receptor pairs between control and IR-PG, which contribute to the signaling from MECs, stromal and endothelial cells to acinar and Etv1+ cells. Dot color reflects communication probabilities and dot size represents computed pvalues. Empty space means the communication probability is zero. pvalues are computed from one-sided permutation test. (F and G) Comparison of the significant ligand-receptor pairs between control and IR-PG, which contribute to the signaling from acinar and Etv1+ cells to immune populations. Dot color reflects communication probabilities and dot size represents computed pvalues. Empty space means the communication probability is zero. pvalues are computed from one-sided permutation test.

Given that too few ligands and receptors were differentially expressed, we next used CellChat, which is sensitive to expression patterns in ligands and receptors themselves, as well as their cofactors, and weighs the size of a given cluster and the proportion of cells within a cluster expressing a gene. CellChat predicted similar ligand-receptor interactions in the IR-PG compared to those from the control glands (Table S3). There was an increase in the number of interactions after IR from 3128 in the control to 3191 in IR PG, although the predicted interaction strength was slightly reduced (Figure 5A). Interactions with *Rpsa*, *Hsp90aa1*, or *Hras* could not be confirmed since these are not included in CellChat’s database of ligand-receptor pairs. CellChat predicted that MECs, stromal, and endothelial cells had the highest number of differential interactions while B-cells and T-cells had the greatest difference in interaction strengths (Figures 5B and S5A). A summary of the number of differential interactions per cell type is provided in Data S1. Here, we focus specifically on acinar and Etv1+ cells.

CellChat predicted altered signaling from MECs, stromal, and endothelial cells to both acinar and Etv1+ cells (Figures 5C and 5D). Generally, this seemed to be predominantly mediated by fewer interactions between *Cd44* and *Sdc4* with multiple collagens and laminins (Figure 5E). We also saw a shift in Neurotrophin signaling interactions after IR, which were decreased between acinar cells and MECs or Stromal cells, but increased with endothelial cells (Figure 5E); this shift also involved changes in the ligand *Ntf5* and the appearance of a more significant contribution from *Ngf-Ntrk1* interactions (Figure S5B). Disruption of neurotrophin signaling was also recently reported in irradiated human salivary glands.^45^ Altered signaling in Etv1+ cells also involved loss of *Igf1-Igf1r* interactions with stromal and endothelial cells, and increased interactions via *Nrg2-Erbb3* with stromal cells (Figure 5E). A key difference between the two cell types was their differential interactions after IR with immune populations. In acinar cells, there were increased interactions with T-cells and B-cells (Figures 5C and 5F), but the strength of the interactions was generally lower (Figure S5C). In contrast, Etv1+ cells showed generally stronger interactions with T-cells, with the exception of FoxP3+ cells, which had fewer interactions (Figures 5D, 5G, and S5C).

These results combined suggest that this model of IR injury shows alterations in cell adhesion interactions with the extracellular matrix (i.e. via Cd44, Sdc4, collagen and laminin), as well as changes in neurotrophin (Ntrk2, Ntf5, Ntf3), neuregulin (Nrg1, Nrg2, Erbb3), and IGF (Igf1, Igf1r) pathways. Furthermore, they suggest active involvement of immune cells, particularly T-cells, in mediating cellular responses after IR, although the specific mechanisms involved are still unclear. Further mechanistic studies are encouraged to determine the functional relevance of these predicted interactions.

## Discussion

We generated a scRNA-seq resource of adult PG that includes a chronic IR injury model. This resource allowed us to identify a discrete cell cluster of secretory cells defined by *Etv1* expression, and to predict putative ligand-receptor interactions that mediate key signaling pathways between secretory cell types and their microenvironment during homeostasis and after injury.

The near exclusivity of *Etv1* expression in a single cluster is intriguing but it is not known whether it represents a cell-type-specific marker or a cell state. Their mixed histological localization suggests the latter. We saw expression of *Etv1* in both acinar and duct compartments, particularly close to the intercalated duct, similar to observations made by Song et al. in adult mouse SMG.^50^ *Etv1* is one of the top transcription factors in the salivary glands^41^ and it was previously reported to be enriched in putative salivary stem cells defined by expression of Lin–CD24+c-Kit+Sca1+.^51^ *Etv1* was also recently associated with the development of the acinar epithelium in the mouse SMG.^28^ These observations may be suggestive of *Etv1* being involved in an intermediate state between intercalated duct and acinar cells. Indeed, experiments in rodents suggest that intercalated duct cells may harbor stem cells that can differentiate into acinar cells or other duct cells.^52^^,^^53^^,^^54^ *Etv1+*cells showed enrichment of *Erbb3* expression, which was predicted to mediate signaling via neuregulin ligands derived from MECs and stromal cells. Erbb3 signaling is critical for SG development and plays a crucial role in organogenesis. Branching morphogenesis in the mouse SMG depends on intraepithelial signaling mediated by ErbB2, ErbB3, and neuregulin 1 (Nrg1).^55^ Nrg1-null embryos show reduced innervation and defective branching morphogenesis.^56^^,^^57^ Thus, it is plausible that *Etv1*+ (*Erbb3+)* cells in the adult parotid gland could be involved in either replenishment of the epithelium or wound healing, and may function as a proacinar population in the PG.

Our finding that the neurotrophic receptor *Ntrk2* is enriched in acinar cells is interesting because of the precedent of using neurotrophic factors such as neurturin to preserve function in irradiated SGs.^37^^,^^58^ Moreover, we recently reported that in humans, IR chronically dysregulates the neurotrophin signaling pathway in both PG and SMG and is associated with functional and morphological abnormalities.^45^ Ligand-receptor analysis predicts that stromal cells and MECs communicate with *Ntrk2-*expressing acinar cells via *Ntf5* and *Ntf3*, respectively. Considering the localization of MECs surrounding acinar cells, it is likely that both juxtracrine and paracrine signaling takes place. The function of the Ntrk2 receptor in salivary acinar cells is not known but the gene is also highly expressed in Neurogenin 3-positive (Ngn3+) endocrine progenitors in the pancreas^59^ and its activation regulates Ngn3+ cell fate commitment. Neurotrophin receptors are also mutated or upregulated in a variety of cancers, suggesting a role in proliferation and differentiation. In the SMG, *Ntrk2* is expressed in serous acinar cells but not in seromucous acinar cells,^28^ indicating that *Ntrk2* signaling may be important for the serous acinar phenotype, which is predominant in the PG. Furthermore, we recently identified that *NTRK2* is highly upregulated in MECs of irradiated human SGs along with other neurotrophin receptors and stimulation of neurotrophin signaling *in vitro* promoted myoepithelial differentiation.^45^ In the lacrimal gland, neurotrophins are expressed in acini while neurotrophin receptors are expressed by MECs,^60^ suggesting that neurotrophin signaling may mediate intercellular communication between acinar cells and MECs in other exocrine tissues. Moreover, given that Ntrk2 is expressed on the cell surface, it may also provide a viable strategy to FACS-sort acinar cells from parotid gland to investigate expansion or differentiation of acinar cells *in vitro.* The latter application would likely require a combination of markers since*Ntrk2* is also expressed in *Etv1*+, myoepithelial and stromal cells.

The IR model used in our study has been previously used to demonstrate the protective effect of neurturin gene-transfer to prevent loss of saliva in the IR-SMG.^37^ In contrast to more severe models of IR injury,^12^^,^^46^^,^^48^^,^^61^^,^^62^ this model does not result in extensive loss of acinar cells and is only mildly fibrotic; nonetheless, secretory function is not regained after IR without therapeutic intervention. This model is ideal for evaluating transcriptional changes that occur in acinar cells which lead to chronic loss of secretion. Indeed, acinar cells had the second largest number of DEGs after IR in this model. Surprisingly, CD4^+^CD8^+^ cells showed the highest number of DEGs after IR, suggesting that chronic damage after IR may be sustained by immunologic mechanisms. There is growing evidence of immune-epithelial interactions in the regulation of tissue homeostasis and wound healing responses with macrophages and regulatory T-cells (T_regs_; FoxP3+) garnering the most attention.^63^ Through Notch-mediated signaling, mammary gland stem cells induced resident macrophages to produce Wnt ligands ultimately leading to mammary stem cell proliferation.^64^ Depletion of T_regs_ in the intestine leads to a reduction in LGR5+ stem cells.^65^ Given the extensive ligand-receptor interactions between *Etv1*+ cells and immune cells, it is interesting to speculate a functional role of *Etv1*+ cells in directing the localization and activation of resident immune populations. In the epidermis, distinct cellular populations around the hair follicle produce distinct chemokines to direct innate immune cell populations.^66^ For example, the interaction between *Etv1*+ and *FoxP3*+ cells via *Cdh1*-*Itgae* (E-cadherin and integrin-α-E) may represent the physical tethering of this sub-population of T-cells to the salivary epithelium under homeostasis,^67^ and it was increased after IR (Figure 5G). It is interesting to note that radiation treatment led to a 1.5-fold increase in T_regs_ without a concomitant change in *Etv1*+ cells or macrophages. Given the extensive role macrophages and *FoxP3*+ cells serve in injury and regeneration models, more work is required to unravel the impact of these T_regs_-epithelial interactions population during SG dysfunction.

Radiation treatment also resulted in the greatest increase in CD4^+^CD8^+^ populations and the most DEGs observed in the CD4^+^CD8^+^ cells (Figure 4). Clinical evaluation of SMG by immunohistochemistry following radiotherapy has revealed increased T-cells (CD3^+^, CD4^+^ or CD8^+^) in the periacinar area and B cell (CD20^+^) nodules in the periductal area.^68^ The DEGs in the CD4^+^CD8^+^ population suggest an imbalance in immune regulation following irradiation. Increases in KLF2 in IR PGs may represent a shift in T-cell populations as KLF2 is highly expressed in naive and memory T-cells and downregulated by TCR activation and cytokine stimulation in effector T-cells.^69^ In addition, high levels of KLF2 inhibit T-cell proliferation and clonal expansion.^69^ KLF6 also inhibits cell proliferation and is co-regulated with KLF2 in MCF-7 cells.^70^ Thus, high levels of KLF2 and KLF6 coupled with a lack of cytokines and chemokines on the DEGs suggest that the increase in CD4^+^CD8^+^T-cells may represent a naive population; however further kinetic analysis is required. This is also supported by a decrease in *Ctla2a*, which encodes for a cysteine protease that serves an immunosuppressive function in retinal pigment epithelium^71^^,^^72^ and promotes the conversion of CD4^+^T cells to Treg cells via Transforming Growth Factor β (TGFβ) signaling.^73^ Lymphotoxin-β (LT-β), encoded by *Ltb*, is a TNF family member cytokine that has been predominantly studied in development and organization of lymphoid tissues.^74^ LT-β can mediate both regeneration and chronic tissue injury in epithelial organs via nuclear factor-κB (NF-κB) pathway.^75^^,^^76^ Blocking the LT-β receptor suppresses immune responses by modulating trafficking mechanisms and disrupts the progression of T1DM in NOD mice.^74^ It is interesting to speculate whether the increased LT-β interactions with *Tnfrsf1a* or *CD40* prevent the clearance of immune populations or maintenance of naive T cells. *Ltb* is induced following oxidative stress^77^ and has been proposed to enable communication between lymphocytes and stromal cells,^75^ findings that are corroborated by this work predicting increased interactions with stromal and immune cell populations after IR (Figure 5).

### Limitations of the study

A caveat of this study is the lack of isolation of basal ducts and peripheral nerve cells during PG dissociation, which were not represented. Similar limitations have been reported in other scRNA-seq studies working with adult tissues, which could potentially be overcome using single nuclei RNA-seq analysis. Furthermore, although multiple biological replicates were used, they were pooled together during dissociation before sequencing, thus, cell proportion changes should be considered with caution.

## STAR★Methods

### Key resources table

**Table:** 

REAGENT or RESOURCE	SOURCE	IDENTIFIER
**Antibodies**		

Anti-Smooth muscle actin-Cy3 conjugate 1:100	Millipore Sigma	CAT# C6198; RRID: AB_476856
Anti-TrkB 80G2 (Ntrk2) 1:100	Cell signaling	CAT# 4607; RRID: AB_2155128
Anti-Nkcc1 1:250	Santa Cruz Biotechnology	CAT# sc-21545; RRID: AB_2188633
Anti-Parotid secretory protein antibody 1:100	Abcam	CAT# ab121028; RRID: AB_10950051
Alexa Fluor® 488 AffiniPure F(ab')₂ Fragment Donkey Anti-Goat IgG (H + L). 1:250	Jackson Immunoresearch Laboratories	CAT# 705-546-147; RRID: AB_2340430
Alexa Fluor® 647 AffiniPure F(ab')₂ Fragment Donkey Anti-Goat IgG (H + L) 1:250	Jackson Immunoresearch Laboratories	CAT# 705-606-147; RRID: AB_2340438
Alexa Fluor® 488 AffiniPure F(ab')₂ Fragment Donkey Anti-Rabbit IgG (H + L) 1:250	Jackson Immunoresearch Laboratories	CAT# 711-546-152; RRID: AB_2340619
Cy™3 AffiniPure F(ab')₂ Fragment Donkey Anti-Rabbit IgG (H + L) 1:250	Jackson Immunoresearch Laboratories	CAT# 711-166-152; RRID: AB_2313568
Cy™3 AffiniPure F(ab')₂ Fragment Donkey Anti-Mouse IgG (H + L) 1:250	Jackson Immunoresearch Laboratories	CAT# 715-165-150; RRID: AB_2340813
DAPI (Dihydrochloride) 1:10000	Millipore Sigma	268298

**Chemicals, peptides, and recombinant proteins**		

Paraformaldehyde (formaldehyde) Aqueous solution	Electron Microscopy Sciences	15710
Xylene substitute	Millipore Sigma	A5597-1GAL
Ethanol 100%	Millipore Sigma	E7023
Ethanol 95%	Millipore Sigma	493538
Acetone	Millipore Sigma	179973
Methanol	Fisher Scientific	A412-4

**Critical commercial assays**		

M.O.M (Mouse on Mouse) Immunodetection Kit	Vector Laboratories	MKB-2213-1

**Deposited data**		

scRNAseq from control and IR-PG	This paper	GEO: GSE223516
Ready-to-use Seurat objects	This paper	https://doi.org/10.6084/m9.figshare.20406219
Code	This paper	Github: https://github.com/chiblya/scRNAseq_PG

**Software and algorithms**		

Beacon Designer	PREMIER Biosoft	N/A
R & R studio	https://rstudio.com/	N/A
FIJI	Schindelin et al.^78^	N/A
Seurat v4	(Hao et al.^39^)	N/A
CellChat 1.6.1	(Jin et al.^44^)	N/A
Photoshop	Adobe	N/A
Excel	Microsoft	N/A
Word	Microsoft	N/A
Powerpoint	Microsoft	N/A
NDP.view2	Hakamatsu	U12388-01

### Resource availability

#### Lead contact

Further information and requests for resources and reagents should be directed to and will be fulfilled by the lead contact, Alejandro Chibly (martinez-chibly.agustin@gene.com).

#### Materials availability

This study did not generate new unique reagents.

### Experimental model and subject details

#### C3H mice and irradiation (IR) treatment

C3H female mice were used for the study and were housed at the NIDCR Veterinary Resource Core in accordance with IACUC guidelines. At 6–10 weeks of age, mice received fractionated IR treatment at 5 × 6 Gy (6 Gy/day for 5 days). Only head and neck area was irradiated by placing each animal in a specially built Lucite jig so the animal could be immobilized without the use of anesthetics. IR treatment was delivered with by an X-Rad 320ix system. The mice were housed in a climate- and light-controlled environment, and allowed free access to food and water for 10 months after -IR before scRNA-seq analysis.

### Method details

#### Single-cell dissociation

Parotid glands from 2 female mice per treatment were dissociated in a 15 mL gentleMACS C tube with 5 mL of digestion enzyme using the human tumor dissociation kit (#130-095-929, Miltenyi Biotech, Auburn CA) in RPMI 1640 w/L-Glutamine (Cell applications, Inc, USA). Cell dissociation was performed in a Miltenyi gentleMACS Octo Dissociator using the manufacturer’s preset 37C_h_TDK_2 program. Following dissociation, 5 mL of RPMI media were added to the dissociated cells and centrifuged at 1100 rpm for 10 min. Cells were resuspended in RPMI 1640 w/L-Glutamine with 5% PenStrep (Gibco, USA) and washed twice with RPMI. Cells were passed through 70 μm filters between centrifugation steps. Single-cell dissociation was confirmed by microscopic examination and cell concentration determined with a Cellometer (Nexcelom Biosciences). Cell concentration was adjusted to 5x10^5^ – 1x10^6^ cells/mlprior to analysis with a 10X genomics Next GEM Chromium controller.

#### Library prep and sequencing

Single-cell RNA-seq library preparation was performed at the NIDCR Genomics and Computational Biology Core using a Chromium Single Cell v3 method (10X Genomics) following the manufacturer’s protocol. Pooled single-cell RNA-seq libraries were sequenced on a NextSeq500 sequencer (Illumina). Cell Ranger Single-Cell Software Suite (10X Genomics) was used for demultiplexing, barcode assignment, and unique molecular identifier (UMI) quantification using the mm10 reference genome (Genome Reference Consortium Mouse Build 38) for read alignment.

#### Computational analysis

Cell Ranger files were imported to SEURAT v3 using R & R Studio software and processed for clustering following their default pipeline. As a quality control measure, cells with fewer than 200 genes were not included in subsequent analyses, and those with >5% of UMIs mapping to mitochondrial genes were defined as non-viable or apoptotic and were also excluded. These metrics were based on our previous scRNAseq analysis of murine SMG.^28^ Normalization and scaling were performed following SEURAT’s default pipeline. Data from control and irradiated glands were bioinformatically integrated prior to assigning cell annotations. ‘Clustree’ package was used to determine an optimal resolution for clustering and the resulting clusters were annotated based on the expression of known cell type markers. Cell-defining genes were determined using the ‘FindAllMarkers’ function which uses a Wilcoxon Rank Sum statistical test for analysis. Only genes with adjusted pvalues <0.05 were considered as cell-defining genes. To identify DEGs between treatments, each population was compared individually using the ‘FindMarkers’ function from SEURAT package.

#### Ligand-receptor analysis

A database of curated ligand-receptor pairs was downloaded from Ramikowski et al. (2015). We used scripted code in R to automate the search for ligand and receptor genes within our dataset and leverage that information against the curated database. Additionally, we used CellChat v.1.6.1 (Jin, S. et al., 2021) to predict significant interactions and their associated pathways. Plots were generated using the ‘circlize’ and complexHeatmap packages in R. The code is available in https://github.com/chiblya/scRNAseq_PG.

#### Immunohistochemistry

PGs were fixed in 4% paraformaldehyde overnight at 4°C and dehydrated with 70% Ethanol prior to paraffin embedding. 5 μm sections were deparaffinized with xylene substitute for 10 min and rehydrated with reverse ethanol gradient for 5 min each. Heat induced antigen retrieval was performed using a microwave maintaining sub-boiling temperature for 10 min in a pH 6.0 Citrate Buffer (#21545, EDM Millipore, Darmstadt, Germany). Sections were washed for 5 min with 0.1% Tween20 (Quality Biological, Inc) in PBS 1X (PBST). M.O.M. (Mouse on Mouse) Immunodetection Kit (Vector Laboratories, Burlingame, CA) was used to block non-specific sites for 1 h at room temperature followed by overnight incubation with primary antibodies at 4°C. Tissue sections were washed 3 times for 5 min each with PBST and incubated in secondary antibodies and nuclear stain (Hoechst (Thermo Fisher Scientific, Marietta, OH)) at room temperature for 1 h. Coverslips were mounted with Fluoro-Gel (Electron Microscopy Sciences, Hatfield, PA), and imaging was performed with a Nikon A1R confocal system.

#### Stitch analysis

Etv1+ cell defining genes from control parotid sample (Table S1) were directly imported into STITCH (http://stitch.embl.de/). For reproducibility, analysis was performed selecting a minimum interaction score of 0.7 and limited to less than 10 interactions.

### Quantification and statistical analysis

Differential expression analysis was performed with SEURAT’s default settings, which use a non-parametric Wilcoxon rank-sum test. Similarly, for prediction of cell-cell communication networks with CellChat, the algorithm uses the Wilcoxon rank-sum test with the significance level of 0.05 for differential expression testing. Then, it uses the law of mass action and a random walk-based network propagation technique for calculating probabilities of cellular networks.

## Data Availability

The single-cell RNAseq libraries were deposited in GEO under accession number GEO: GSE223516. The code used for analysis is available in github: https://github.com/chiblya/scRNAseq_PG. All package versions are reported in the gitgub repository. The software used for analysis is described in the key resources table. Ready-to- use Seurat objects are also available via figshare: https://doi.org/10.6084/m9.figshare.20406219. Any additional information required to reanalyze the data reported in this paper is available from the lead contact upon request.

## Supplemental information

Document S1. Figures S1–S5

Table S1. Gene lists, related to Figure 1

Table S2. Ligand-Receptor pairs, related to Figure 3

Table S3. Cell Chat results (control PG), related to Figures 3,4 and 5

Table S4. Differentially expressed genes, related to Figure 4

Table S5. Differentially expressed ligands and receptors, related to Figure 4

Data S1. Cell Chat chord plots, related to Figure 5

## References

[1] Maruyama C.L., Monroe M.M., Hunt J.P., Buchmann L., Baker O.J. (2019). Comparing human and mouse salivary glands: a practice guide for salivary researchers. Oral Dis..

[2] Henriksson R., Fröjd O., Gustafsson H., Johansson S., Yi-Qing C., Franzén L., Bjermer L. (1994). Increase in mast cells and hyaluronic acid correlates to radiation-induced damage and loss of serous acinar cells in salivary glands: the parotid and submandibular glands differ in radiation sensitivity. Br. J. Cancer.

[3] Nagler R.M. (2002). The enigmatic mechanism of irradiation-induced damage to the major salivary glands. Oral Dis..

[4] Gao X., Oei M.S., Ovitt C.E., Sincan M., Melvin J.E. (2018). Transcriptional profiling reveals gland-specific differential expression in the three major salivary glands of the adult mouse. Physiol. Genomics.

[5] Jensen S.B., Pedersen A.M.L., Vissink A., Andersen E., Brown C.G., Davies A.N., Dutilh J., Fulton J.S., Jankovic L., Lopes N.N.F. (2010). A systematic review of salivary gland hypofunction and xerostomia induced by cancer therapies: prevalence, severity and impact on quality of life. Support. Care Cancer.

[6] Tasaka S., Jingu K., Takahashi N., Umezawa R., Yamamoto T., Ishikawa Y., Takeda K., Suzuki Y., Kadoya N. (2021). The long-term recovery of parotid glands in nasopharyngeal carcinoma treated by intensity-modulated radiotherapy. Front. Oncol..

[7] Jensen S.B., Vissink A., Limesand K.H., Reyland M.E. (2019). Salivary gland hypofunction and xerostomia in head and neck radiation patients. J. Natl. Cancer Inst. Monogr..

[8] Eisbruch A., Dawson L.A., Kim H.M., Bradford C.R., Terrell J.E., Chepeha D.B., Teknos T.N., Anzai Y., Marsh L.H., Martel M.K. (1999). Conformal and intensity modulated irradiation of head and neck cancer: the potential for improved target irradiation, salivary gland function, and quality of life. Acta Otorhinolaryngol. Belg..

[9] Henson B.S., Eisbruch A., D’Hondt E., Ship J.A. (1999). Two-year longitudinal study of parotid salivary flow rates in head and neck cancer patients receiving unilateral neck parotid-sparing radiotherapy treatment. Oral Oncol..

[10] Avila J.L., Grundmann O., Burd R., Limesand K.H. (2009). Radiation-induced salivary gland dysfunction results from p53-dependent apoptosis. Int. J. Radiat. Oncol. Biol. Phys..

[11] Meyer S., Chibly A.M., Burd R., Limesand K.H. (2017). Insulin-like growth factor-1-mediated DNA repair in irradiated salivary glands is sirtuin-1 dependent. J. Dent. Res..

[12] Liu X., Cotrim A., Teos L., Zheng C., Swaim W., Mitchell J., Mori Y., Ambudkar I. (2013). Loss of TRPM2 function protects against irradiation-induced salivary gland dysfunction. Nat. Commun..

[13] Gilman K.E., Camden J.M., Klein R.R., Zhang Q., Weisman G.A., Limesand K.H. (2019). P2X7 receptor deletion suppresses gamma-radiation-induced hyposalivation. Am. J. Physiol. Regul. Integr. Comp. Physiol..

[14] Jasmer K.J., Gilman K.E., Muñoz Forti K., Weisman G.A., Limesand K.H. (2020). Radiation-induced salivary gland dysfunction: mechanisms, therapeutics and future directions. J. Clin. Med..

[15] Grundmann O., Mitchell G.C., Limesand K.H. (2009). Sensitivity of salivary glands to radiation: from animal models to therapies. J. Dent. Res..

[16] Dirix P., Nuyts S., Van den Bogaert W. (2006). Radiation-induced xerostomia in patients with head and neck cancer: a literature review. Cancer.

[17] Radfar L., Sirois D.A. (2003). Structural and functional injury in minipig salivary glands following fractionated exposure to 70 Gy of ionizing radiation: an animal model for human radiation-induced salivary gland injury. Oral Surg. Oral Med. Oral Pathol. Oral Radiol. Endod..

[18] Li Y., Taylor J.M.G., Ten Haken R.K., Eisbruch A. (2007). The impact of dose on parotid salivary recovery in head and neck cancer patients treated with radiation therapy. Int. J. Radiat. Oncol. Biol. Phys..

[19] Vissink A., Mitchell J.B., Baum B.J., Limesand K.H., Jensen S.B., Fox P.C., Elting L.S., Langendijk J.A., Coppes R.P., Reyland M.E. (2010). Clinical management of salivary gland hypofunction and xerostomia in head-and-neck cancer patients: successes and barriers. Int. J. Radiat. Oncol. Biol. Phys..

[20] Grün D., van Oudenaarden A. (2015). Design and analysis of single-cell sequencing experiments. Cell.

[21] Kolodziejczyk A.A., Kim J.K., Svensson V., Marioni J.C., Teichmann S.A. (2015). The technology and biology of single-cell RNA sequencing. Mol. Cell.

[22] Trapnell C. (2015). Defining cell types and states with single-cell genomics. Genome Res..

[23] Wang Y., Navin N.E. (2015). Advances and applications of single-cell sequencing technologies. Mol. Cell.

[24] Tabula Muris Consortium, Supplemental text writing group, Principal investigators, Overall coordination, Logistical coordination, Organ collection and processing, Library preparation and sequencing, Computational data analysis, Cell type annotation, Writing group (2018). Single-cell transcriptomics of 20 mouse organs creates a Tabula Muris. Nature.

[25] Jones R.C., Karkanias J., Krasnow M.A., Pisco A.O., Quake S.R., Salzman J., Yosef N., Bulthaup B., Brown P., Tabula Sapiens Consortium∗ (2022). The Tabula Sapiens: a multiple-organ, single-cell transcriptomic atlas of humans. Science.

[26] Chen M., Lin W., Gan J., Lu W., Wang M., Wang X., Yi J., Zhao Z. (2022). Transcriptomic mapping of human parotid gland at single-cell resolution. J. Dent. Res..

[27] Huang N., Pérez P., Kato T., Mikami Y., Okuda K., Gilmore R.C., Conde C.D., Gasmi B., Stein S., Beach M. (2021). SARS-CoV-2 infection of the oral cavity and saliva. Nat. Med..

[28] Hauser B.R., Aure M.H., Kelly M.C., Hoffman M.P., Chibly A.M., Genomics and Computational Biology Core (2020). Generation of a single-cell RNAseq atlas of murine salivary gland development. iScience.

[29] Oyelakin A., Song E.A.C., Min S., Bard J.E., Kann J.V., Horeth E., Smalley K., Kramer J.M., Sinha S., Romano R.A. (2019). Transcriptomic and single-cell analysis of the murine parotid gland. J. Dent. Res..

[30] Sekiguchi R., Martin D., Yamada K.M., Genomics and Computational Biology Core (2020). Single-cell RNA-seq identifies cell diversity in embryonic salivary glands. J. Dent. Res..

[31] Praktiknjo S.D., Obermayer B., Zhu Q., Fang L., Liu H., Quinn H., Stoeckius M., Kocks C., Birchmeier W., Rajewsky N. (2020). Tracing tumorigenesis in a solid tumor model at single-cell resolution. Nat. Commun..

[32] Horeth E., Oyelakin A., Song E.A.C., Che M., Bard J., Min S., Kiripolsky J., Kramer J.M., Sinha S., Romano R.A. (2021). Transcriptomic and single-cell analysis reveals regulatory networks and cellular heterogeneity in mouse primary sjogren's syndrome salivary glands. Front. Immunol..

[33] Hong X., Meng S., Tang D., Wang T., Ding L., Yu H., Li H., Liu D., Dai Y., Yang M. (2020). Single-cell RNA sequencing reveals the expansion of cytotoxic CD4(+) T lymphocytes and a landscape of immune cells in primary sjogren's syndrome. Front. Immunol..

[34] Xu Y., Feng S., Peng Q., Zhu W., Zu Q., Yao X., Zhang Q., Cao J., Jiao Y. (2021). Single-cell RNA sequencing reveals the cell landscape of a radiation-induced liver injury mouse model. Radiat. Med. Prot..

[35] Mukherjee A., Epperly M.W., Shields D., Hou W., Fisher R., Hamade D., Wang H., Saiful Huq M., Bao R., Tabib T. (2021). Ionizing irradiation-induced Fgr in senescent cells mediates fibrosis. Cell Death Discov..

[36] Paldor M., Levkovitch-Siany O., Eidelshtein D., Adar R., Enk C.D., Marmary Y., Elgavish S., Nevo Y., Benyamini H., Plaschkes I. (2022). Single-cell transcriptomics reveals a senescence-associated IL-6/CCR6 axis driving radiodermatitis. EMBO Mol. Med..

[37] Ferreira J.N.A., Zheng C., Lombaert I.M.A., Goldsmith C.M., Cotrim A.P., Symonds J.M., Patel V.N., Hoffman M.P. (2018). Neurturin gene therapy protects parasympathetic function to prevent irradiation-induced murine salivary gland hypofunction. Mol. Ther. Methods Clin. Dev..

[38] Stuart T., Butler A., Hoffman P., Hafemeister C., Papalexi E., Mauck W.M., Hao Y., Stoeckius M., Smibert P., Satija R. (2019). Comprehensive integration of single-cell data. Cell.

[39] Hao Y., Hao S., Andersen-Nissen E., Mauck W.M., Zheng S., Butler A., Lee M.J., Wilk A.J., Darby C., Zager M. (2021). Integrated analysis of multimodal single-cell data. Cell.

[40] Zappia L., Oshlack A. (2018). Clustering trees: a visualization for evaluating clusterings at multiple resolutions. Gigascience.

[41] Michael D.G., Pranzatelli T.J.F., Warner B.M., Yin H., Chiorini J.A. (2019). Integrated epigenetic mapping of human and mouse salivary gene regulation. J. Dent. Res..

[42] Kuhn M., von Mering C., Campillos M., Jensen L.J., Bork P. (2008). STITCH: interaction networks of chemicals and proteins. Nucleic Acids Res..

[43] Ramilowski J.A., Goldberg T., Harshbarger J., Kloppmann E., Lizio M., Satagopam V.P., Itoh M., Kawaji H., Carninci P., Rost B., Forrest A.R.R. (2015). A draft network of ligand-receptor-mediated multicellular signalling in human. Nat. Commun..

[44] Jin S., Guerrero-Juarez C.F., Zhang L., Chang I., Ramos R., Kuan C.H., Myung P., Plikus M.V., Nie Q. (2021). Inference and analysis of cell-cell communication using CellChat. Nat. Commun..

[45] Chibly A.M., Patel V.N., Aure M.H., Pasquale M.C., Martin G.E., Ghannam M., Andrade J., Denegre N.G., Simpson C., NIDCD/NIDCR Genomics and Computational Biology Core (2023). Neurotrophin signaling is a central mechanism of salivary dysfunction after irradiation that disrupts myoepithelial cells. NPJ Regen. Med..

[46] Teos L.Y., Zheng C.Y., Liu X., Swaim W.D., Goldsmith C.M., Cotrim A.P., Baum B.J., Ambudkar I.S. (2016). Adenovirus-mediated hAQP1 expression in irradiated mouse salivary glands causes recovery of saliva secretion by enhancing acinar cell volume decrease. Gene Ther..

[47] Zheng C., Cotrim A.P., Rowzee A., Swaim W., Sowers A., Mitchell J.B., Baum B.J. (2011). Prevention of radiation-induced salivary hypofunction following hKGF gene delivery to murine submandibular glands. Clin. Cancer Res..

[48] Lombaert I.M.A., Brunsting J.F., Wierenga P.K., Kampinga H.H., de Haan G., Coppes R.P. (2008). Keratinocyte growth factor prevents radiation damage to salivary glands by expansion of the stem/progenitor pool. Stem Cell..

[49] TheGeneOntologyConsortium (2019). The gene ontology resource: 20 years and still GOing strong. Nucleic Acids Res..

[50] Song E.A.C., Smalley K., Oyelakin A., Horeth E., Che M., Wrynn T., Osinski J., Romano R.A., Sinha S. (2023). Genetic study of Elf5 and ehf in the mouse salivary gland. J. Dent. Res..

[51] Xiao N., Lin Y., Cao H., Sirjani D., Giaccia A.J., Koong A.C., Kong C.S., Diehn M., Le Q.T. (2014). Neurotrophic factor GDNF promotes survival of salivary stem cells. J. Clin. Invest..

[52] Katsumata O., Sato Y.I., Sakai Y., Yamashina S. (2009). Intercalated duct cells in the rat parotid gland may behave as tissue stem cells. Anat. Sci. Int..

[53] Denny P.C., Liu P., Denny P.A. (1999). Evidence of a phenotypically determined ductal cell lineage in mouse salivary glands. Anat. Rec..

[54] Denny P.C., Ball W.D., Redman R.S. (1997). Salivary glands: a paradigm for diversity of gland development. Crit. Rev. Oral Biol. Med..

[55] Miyazaki Y., Nakanishi Y., Hieda Y. (2004). Tissue interaction mediated by neuregulin-1 and ErbB receptors regulates epithelial morphogenesis of mouse embryonic submandibular gland. Dev. Dyn..

[56] Nedvetsky P.I., Emmerson E., Finley J.K., Ettinger A., Cruz-Pacheco N., Prochazka J., Haddox C.L., Northrup E., Hodges C., Mostov K.E. (2014). Parasympathetic innervation regulates tubulogenesis in the developing salivary gland. Dev. Cell.

[57] Mattingly A., Finley J.K., Knox S.M. (2015). Salivary gland development and disease. Wiley Interdiscip. Rev. Dev. Biol..

[58] Lombaert I.M.A., Patel V.N., Jones C.E., Villier D.C., Canada A.E., Moore M.R., Berenstein E., Zheng C., Goldsmith C.M., Chorini J.A. (2020). CERE-120 prevents irradiation-induced hypofunction and restores immune homeostasis in porcine salivary glands. Mol. Ther. Methods Clin. Dev..

[59] Shamblott M.J., O’Driscoll M.L., Gomez D.L., McGuire D.L. (2016). Neurogenin 3 is regulated by neurotrophic tyrosine kinase receptor type 2 (TRKB) signaling in the adult human exocrine pancreas. Cell Commun. Signal..

[60] Ghinelli E., Johansson J., Ríos J.D., Chen L.-L., Zoukhri D., Hodges R.R., Dartt D.A. (2003). Presence and localization of neurotrophins and neurotrophin receptors in rat lacrimal gland. Invest. Ophthalmol. Vis. Sci..

[61] Lombaert I.M.A., Brunsting J.F., Wierenga P.K., Faber H., Stokman M.A., Kok T., Visser W.H., Kampinga H.H., de Haan G., Coppes R.P. (2008). Rescue of salivary gland function after stem cell transplantation in irradiated glands. PLoS One.

[62] Ninche N., Kwak M., Ghazizadeh S. (2020). Diverse epithelial cell populations contribute to the regeneration of secretory units in injured salivary glands. Development.

[63] Naik S., Larsen S.B., Cowley C.J., Fuchs E. (2018). Two to tango: dialog between immunity and stem cells in health and disease. Cell.

[64] Chakrabarti R., Celià-Terrassa T., Kumar S., Hang X., Wei Y., Choudhury A., Hwang J., Peng J., Nixon B., Grady J.J. (2018). Notch ligand Dll1 mediates cross-talk between mammary stem cells and the macrophageal niche. Science.

[65] Biton M., Haber A.L., Rogel N., Burgin G., Beyaz S., Schnell A., Ashenberg O., Su C.-W., Smillie C., Shekhar K. (2018). T helper cell cytokines modulate intestinal stem cell renewal and differentiation. Cell.

[66] Mansfield K., Naik S. (2020). Unraveling immune-epithelial interactions in skin homeostasis and injury. Yale J. Biol. Med..

[67] Agace W.W., Higgins J.M., Sadasivan B., Brenner M.B., Parker C.M. (2000). T-lymphocyte-epithelial-cell interactions: integrin alpha(E)(CD103)beta(7), LEEP-CAM and chemokines. Curr. Opin. Cell Biol..

[68] Teymoortash A., Simolka N., Schrader C., Tiemann M., Werner J.A. (2005). Lymphocyte subsets in irradiation-induced sialadenitis of the submandibular gland. Histopathology.

[69] Preston G.C., Feijoo-Carnero C., Schurch N., Cowling V.H., Cantrell D.A. (2013). The impact of KLF2 modulation on the transcriptional Program and function of CD8 T cells. PLoS One.

[70] Ebert R., Zeck S., Meissner-Weigl J., Klotz B., Rachner T.D., Benad P., Klein-Hitpass L., Rudert M., Hofbauer L.C., Jakob F. (2012). Krüppel-like factors KLF2 and 6 and Ki-67 are direct targets of zoledronic acid in MCF-7 cells. Bone.

[71] Sugita S., Horie S., Nakamura O., Maruyama K., Takase H., Usui Y., Takeuchi M., Ishidoh K., Koike M., Uchiyama Y. (2009). Acquisition of T Regulatory function in cathepsin L-inhibited T cells by eye-derived CTLA-2α during inflammatory conditions. J. Immunol..

[72] Sugita S., Horie S., Nakamura O., Futagami Y., Takase H., Keino H., Aburatani H., Katunuma N., Ishidoh K., Yamamoto Y., Mochizuki M. (2008). Retinal pigment epithelium-derived CTLA-2α induces TGFβ-producing T regulatory cells. J. Immunol..

[73] Sugita S., Yamada Y., Horie S., Nakamura O., Ishidoh K., Yamamoto Y., Yamagami S., Mochizuki M. (2011). Induction of T Regulatory cells by cytotoxic T-lymphocyte antigen-2α on corneal endothelial cells. Invest. Ophthalmol. Vis. Sci..

[74] McCarthy D.D., Summers-Deluca L., Vu F., Chiu S., Gao Y., Gommerman J.L. (2006). The lymphotoxin pathway. Immunol. Res..

[75] Wolf M.J., Seleznik G.M., Zeller N., Heikenwalder M. (2010). The unexpected role of lymphotoxin β receptor signaling in carcinogenesis: from lymphoid tissue formation to liver and prostate cancer development. Oncogene.

[76] Tumanov A.V., Koroleva E.P., Christiansen P.A., Khan M.A., Ruddy M.J., Burnette B., Papa S., Franzoso G., Nedospasov S.A., Fu Y.X., Anders R.A. (2009). T cell-derived lymphotoxin regulates liver regeneration. Gastroenterology.

[77] Wong G.H. (1995). Protective roles of cytokines against radiation: induction of mitochondrial MnSOD. Biochim. Biophys. Acta.

[78] Schindelin J., Arganda-Carreras I., Frise E. (2012). Fiji: an open-source platform for biological-image analysis. Nat. Methods.

