# Supplementary material for: Evaluating the transcriptional landscape and cell-cell communication networks in chronically irradiated parotid glands
Source: iScience. 2023 Apr 11;26(5):106660. doi: 10.1016/j.isci.2023.106660 (PMC10165028; doi:10.1016/j.isci.2023.106660)
Supplement: Document S1. Figures S1–S5 [file mmc1.pdf]

iScience, Volume *26*

Supplemental information

Evaluating the transcriptional landscape and cell-cell communication networks in chronically irradiated parotid glands

Brenna A. Rheinheimer, Mary C. Pasquale, NIDCD/NIDCR Genomics and Computational Biology Core, Kirsten H. Limesand, Matthew P. Hoffman, and Alejandro M. Chibly

Figure S1. Annotation strategy, Related to Figure 1

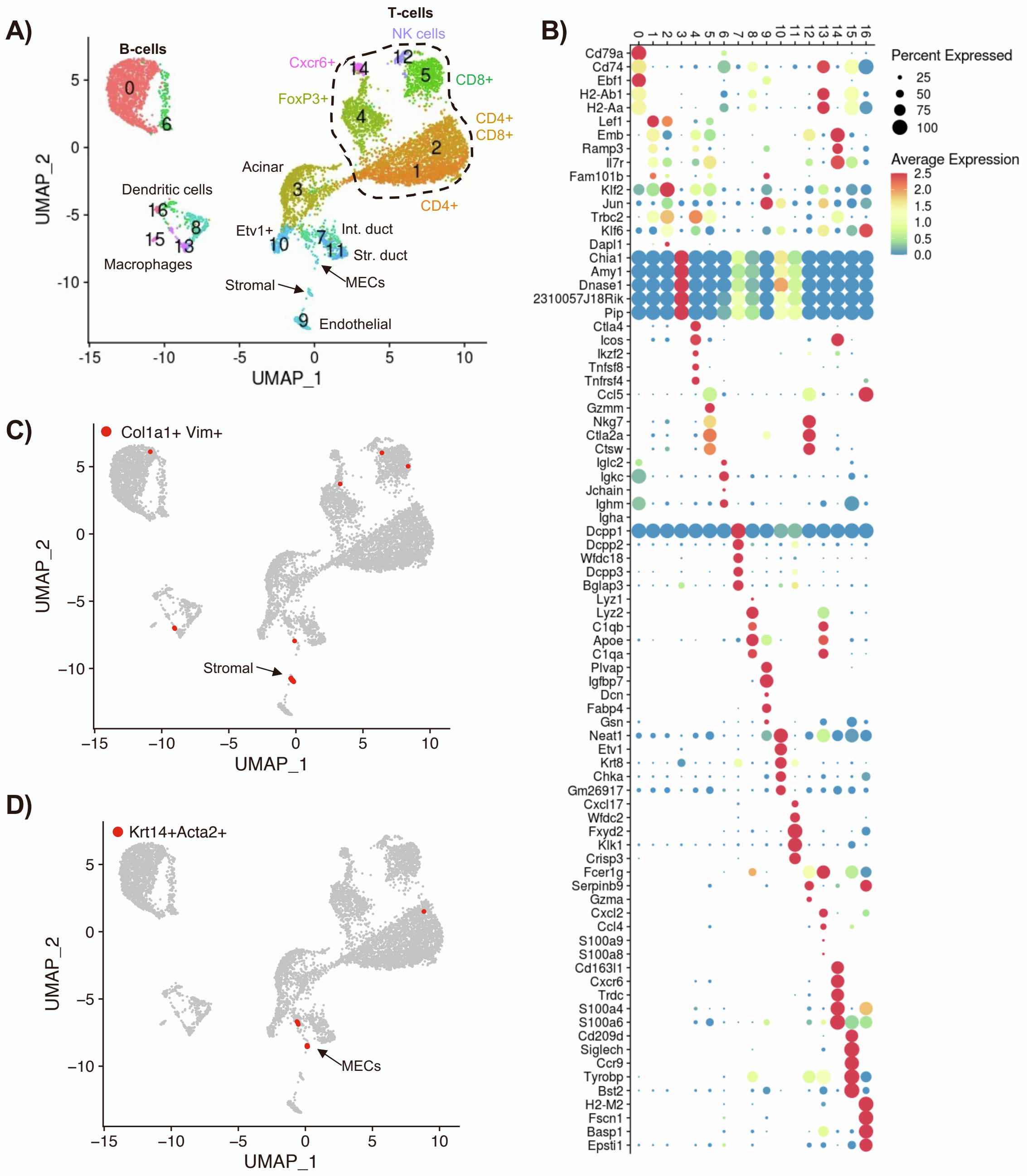

Figure S1: Annotation strategy. Related to Figure 1

A) Unsupervised clustering of integrated control and irradiated mouse parotid gland (n=1 per treatment)

B) Balloon plot of top cluster-defining genes. Color is relative to scaled gene expression and size of the dot represents the percentage of cells within a cluster expressing the gene

C) UMAP highlighting cells that express the stromal markers Col1a1 and Vim

D) UMAP highlighting myoepithelial cells that express Krt14 and Acta2

Figure S2. Secretory cells, Related to Figure 2

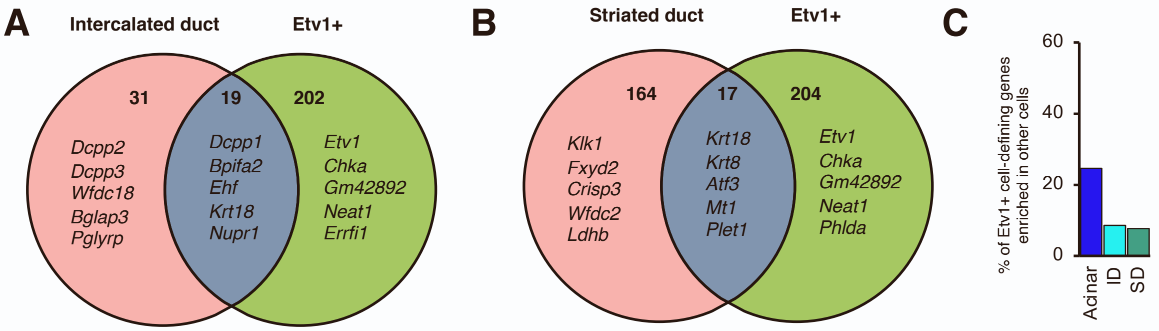

D

GO Biological Processes (Acinar cells)			
**Pathway ID**	**Pathway description**	**Gene count**	**FDR**
GO.0002018	renin-angiotensin regulation of aldosterone production	3	0.000359
GO.0003081	regulation of systemic arterial blood pressure by renin-angiotensin	5	0.000359
GO.0003073	regulation of systemic arterial blood pressure	6	0.00163
GO.0002035	brain renin-angiotensin system	3	0.00227
GO.0050708	regulation of protein secretion	9	0.00476
**KEGG pathways (Acinar cells)**			
**Pathway ID**	**Pathway description**	**Gene count**	**FDR**
4614	Renin-angiotensin system	5	2.21E-06
4150	mTOR signaling pathway	5	0.000499
4970	Salivary secretion	5	0.000867

Figure S2. **Secretory cells. Related to Figure 2**

A) Venn diagram comparing defining genes for *Etv1*+ and ID populations. The numbers in the left and right panels indicates the number of unique genes in the corresponding population whereas the number in the central panel reflects the overlap between the two populations.
B) Venn diagram comparing defining genes for *Etv1*+ and SD populations. The numbers in the left and right panels indicates the number of unique genes in the corresponding population whereas the number in the central panel reflects the overlap between the two populations.
C) Bar graph with percentage of Etv1+ defining genes enriched in other epithelial cells.
D) Results from STITCH analysis showing top biological processes and KEGG pathways associated with defining-genes from acinar cells.

Figure S3. Ligand-receptor analysis of secretory cells, Related to Figure 3

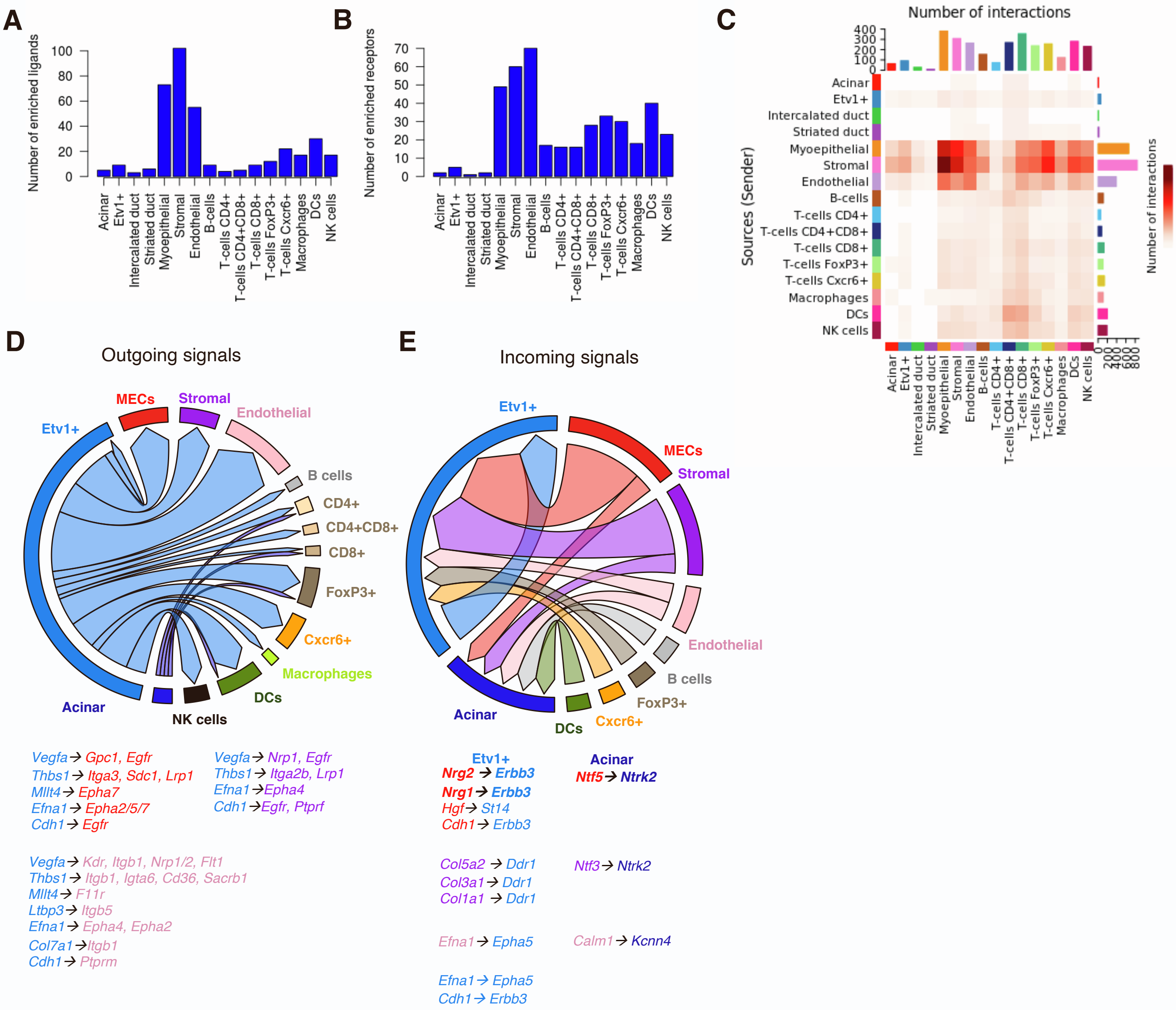

Figure S3. Ligand-receptor analysis of secretory cells. Related to Figure 3.

A-B) Bar graphs with number of identified ligands and receptors among cell-defining genes from all populations.
C) Heatmap of possible interactions between any two cell populations. Red (positive values) in the color bar indicate higher number of predicted interactions.
D-E) Chord plot summarizing putative ligand-receptor interactions with Etv1+ cell ligands. The arrows point to the cell expressing the corresponding receptors and are color-coded based on the source of the ligand. The thickness of the arrow is relative to the number of putative pairs identified between Etv1 cells and the cell type pointed by the arrow. Representative ligand-receptor pairs are shown beside the chord plots.

Figure S4. Cell-specific IR-induced DEGs, Related to Figure 4

A

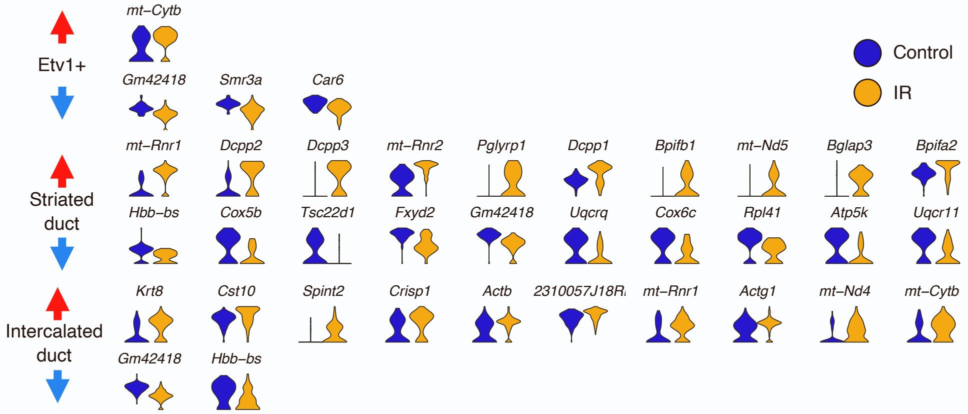

B

GO biological process complete (IR-induced DEGs in acinar cells)	FDR	Genes
regulation of transepithelial transport (GO:0150111)	1.73E-02	Actb, Actg1
electron transport coupled proton transport (GO:0015990)	2.26E-02	mt-Cyb, Mtnd4
energy coupled proton transmembrane transport, against electrochemical gradient (GO:0015988)	2.23E-02	mt-Cyb, Mtnd4
positive regulation of intrinsic apoptotic signaling pathway by p53 class mediator (GO:1902255)	5.35E-04	Ubb, Rpl11, Rps7, Pppia, Rps3, Tpt1, Rack1
regulation of endodeoxyribonuclease activity (GO:0032071)	3.75E-02	Npm1, Rps3
cytoplasmic translation (GO:0002181)	6.15E-15	Rps and Rpl genes
positive regulation of signal transduction by p53 class mediator (GO:1901798)	1.67E-04	Ubb, Rpl11, Rps7, Ddx5

GO biological process complete (IR-induced DEGs in CD4+CD8+ T –cells)	FDR	Genes
T cell receptor V(D)J recombination (GO:0033153)	3.39E-02	Bcl11b, Tcf7
chaperone-mediated protein complex assembly (GO:0051131)	1.45E-02	Hspd1, Hsp90aa1, Hsp90ab1
lymphocyte differentiation (GO:0030098)	5.47E-03	Zfp36l2, Tcf7, Ptpn6, Rhoh, Cd28, Klf6, Gpr183
leukocyte differentiation (GO:0002521)	1.43E-03	Zfp36l2, Tcf7, Junb, Rhoh, Cd28, Klf6, Gpr183, Bcl11b, Jun,
apoptotic signaling pathway (GO:0097190)	3.99E-02	Shisa5, Prkca, Ppp1r15a, Jun, Crip1, Cd28
axonogenesis (GO:0007409)	9.62E-03	Hsp90aa1, Prkca, Emb, Actb, Bcl11b, Evl, Rhoh, Hsp90ab1
regulation of ERK1 and ERK2 cascade (GO:0070372)	4.48E-02	Prkca, Jun, Dusp1, Ptpn6, Gpr183, Ramp3

Figure S4. Cell-specific IR-induced DEGs. Related to Figure 4.

A) Violin plots of top 10 (if present) up and downregulated genes in epithelial populations. Red and blue arrows denote upregulated and downregulated genes, respectively.

B) Representative output from gene ontology analysis with IR-induced DEGs in acinar and CD4+CD8+ T-cells showing dysregulated processes and their associated genes.

Figure S5. Dysregulated ligand-receptor pairs post-IR, Related to Figure 5

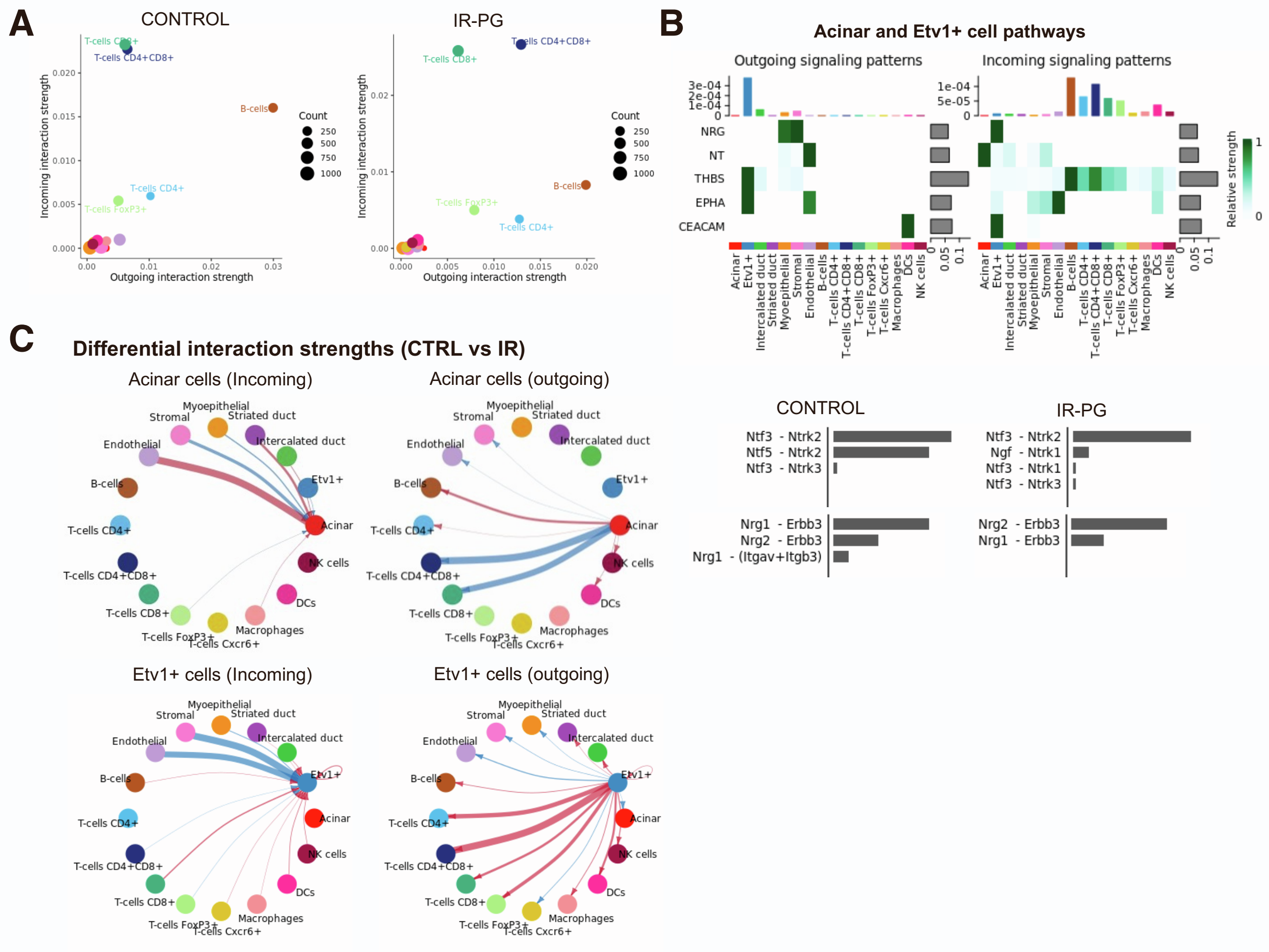

Figure S5. Dysregulated ligand-receptor pairs post-IR. Related to Figure 5.

A) 2D representation of incoming vs outgoing interaction strengths for all cell types relative to each other.

B) Heatmap shows the relative importance of each cell group in IR-PG based on CellChat-computed network centrality measures of NRG, NT, THBS, EPHA, and CEACAM signaling networks. Relative contribution of each ligand-receptor pair to the overall communication network of NRG and NT signaling pathways, which is the ratio of the total communication probability of the inferred network of each ligand-receptor pair to that of the signaling pathway.

C) Differential interactions strength between acinar and Etv1+ populations with all other cell types in IR-PG compared to control. Red (positive values) and blue (negative values) in the color bar indicate higher number of predicted interactions in IR-PG and controls, respectively
