# Supplementary material for: Evaluating the transcriptional landscape and cell-cell communication networks in chronically irradiated parotid glands
Source: iScience. 2023 Apr 11;26(5):106660. doi: 10.1016/j.isci.2023.106660 (PMC10165028; doi:10.1016/j.isci.2023.106660)

Supplemental Data S1. Cell Chat chord plots. Related to Figure 5

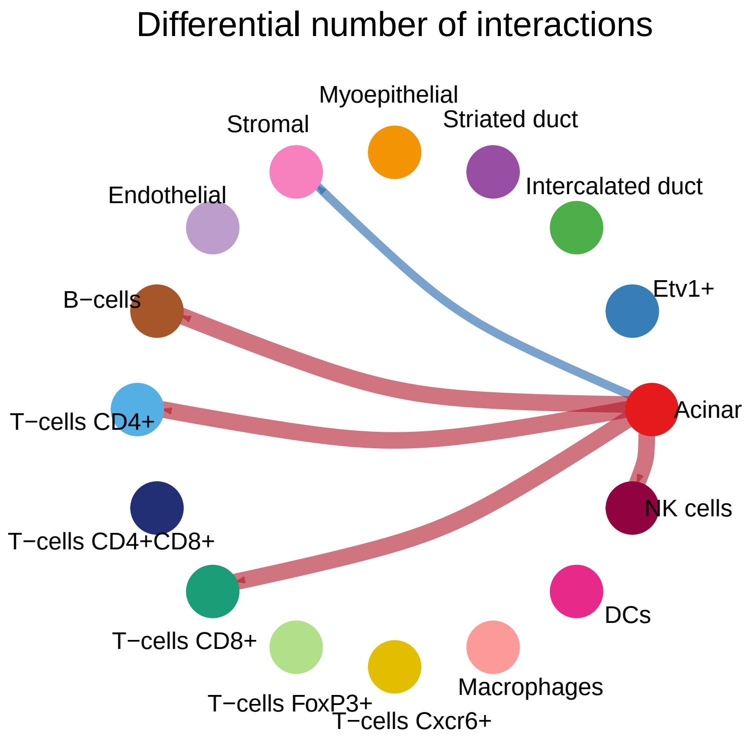

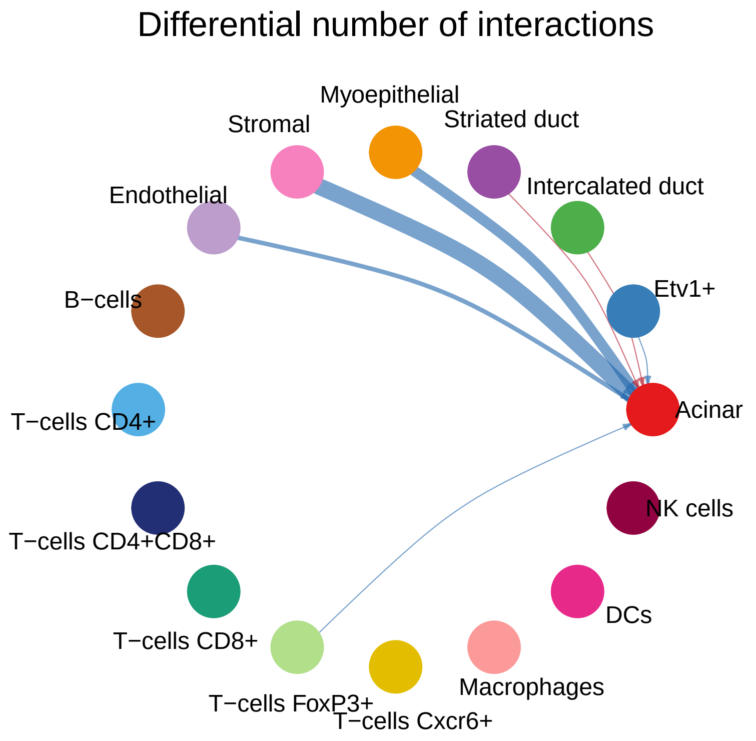

Supplemental Data S1. Cell Chat chord plots. Related to Figure 5

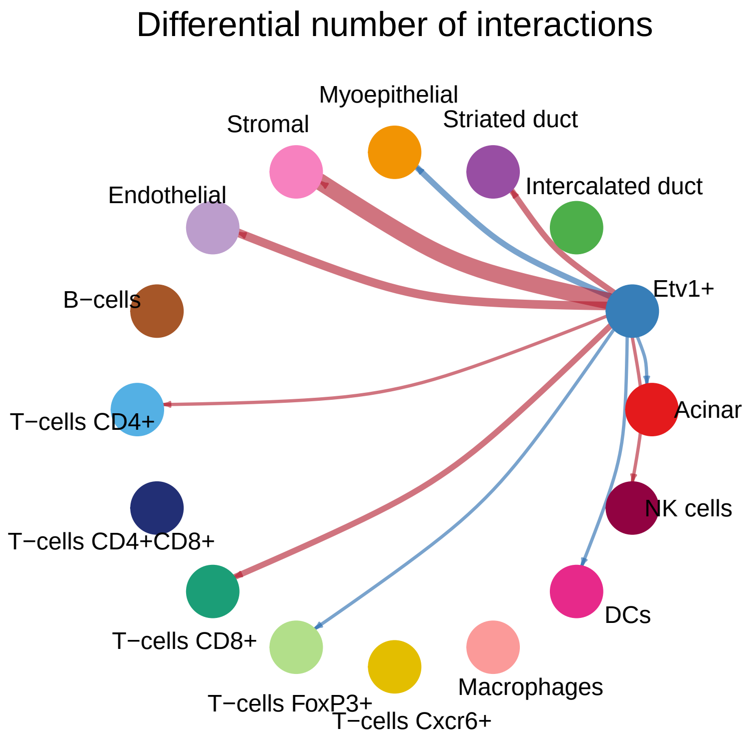

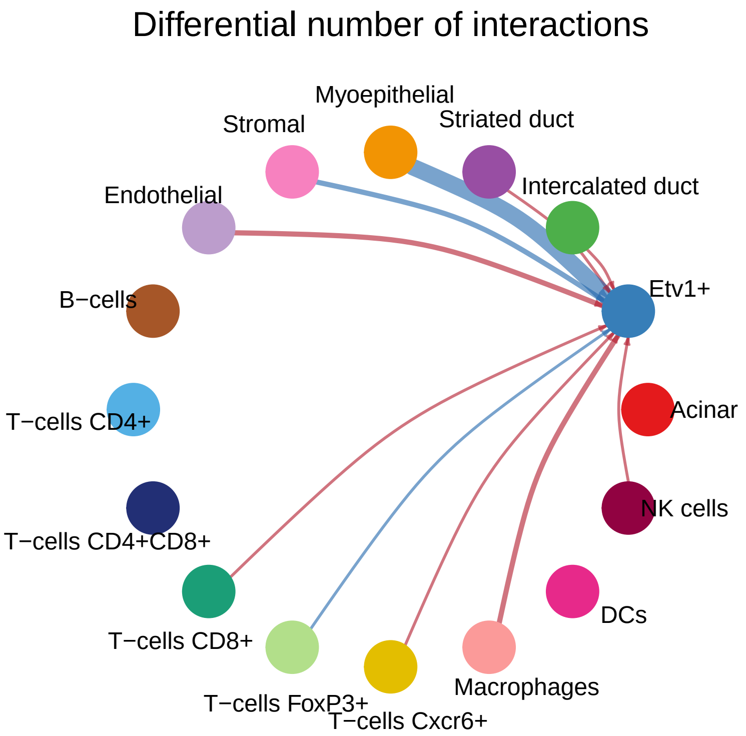

Supplemental Data S1. Cell Chat chord plots. Related to Figure 5

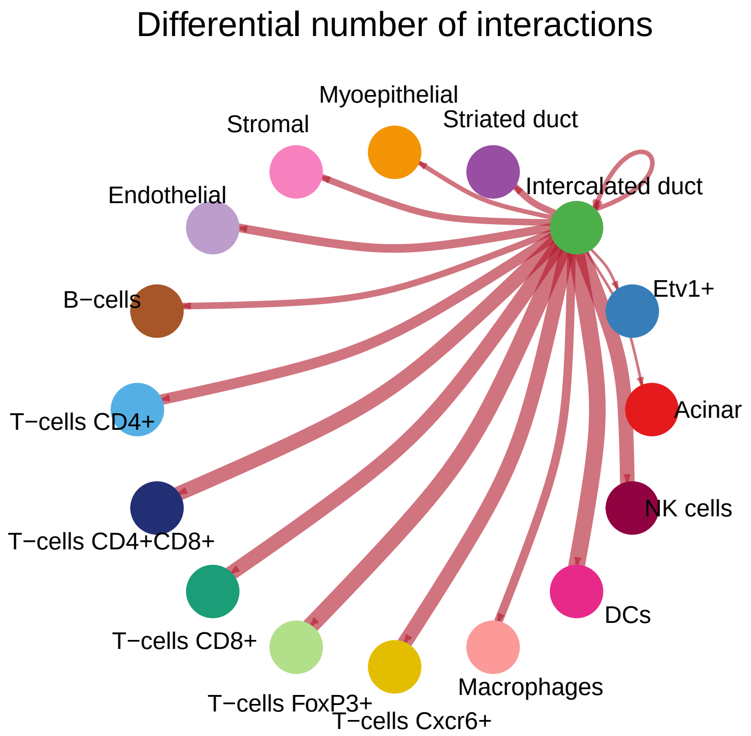

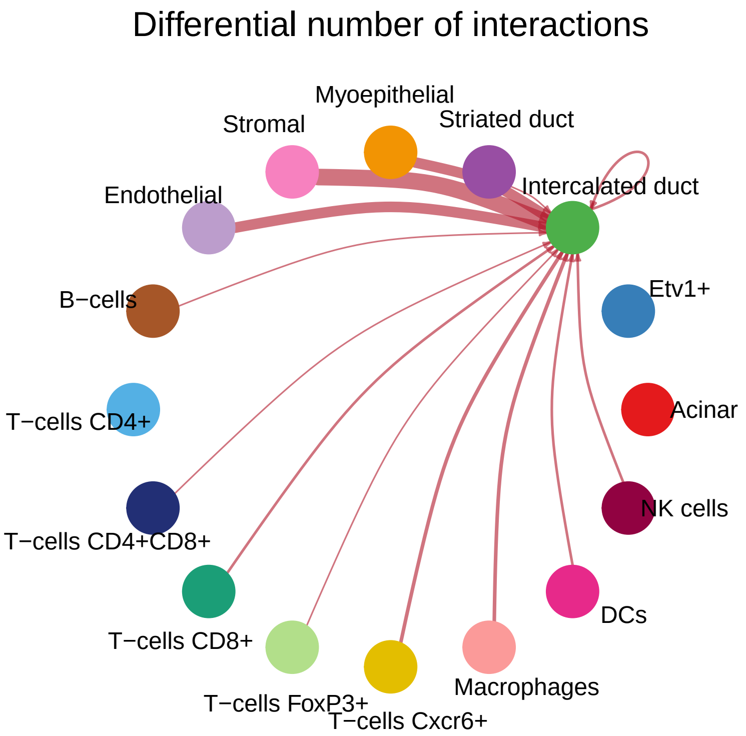

Supplemental Data S1. Cell Chat chord plots. Related to Figure 5

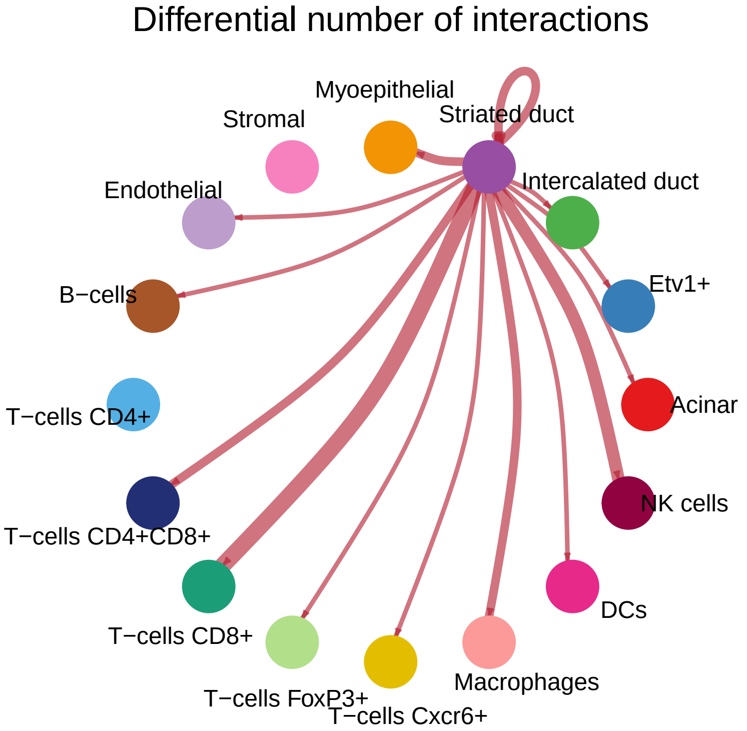

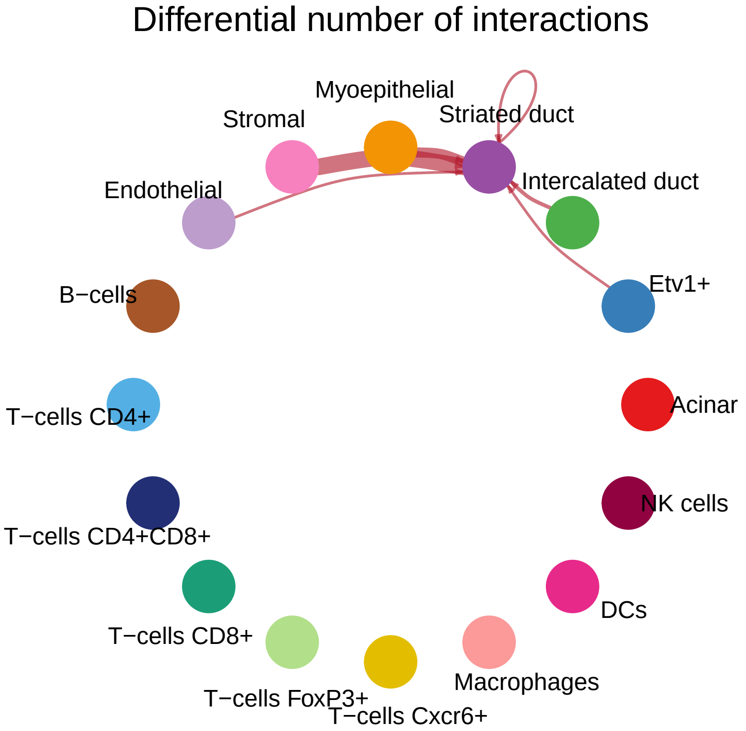

Supplemental Data S1. Cell Chat chord plots. Related to Figure 5

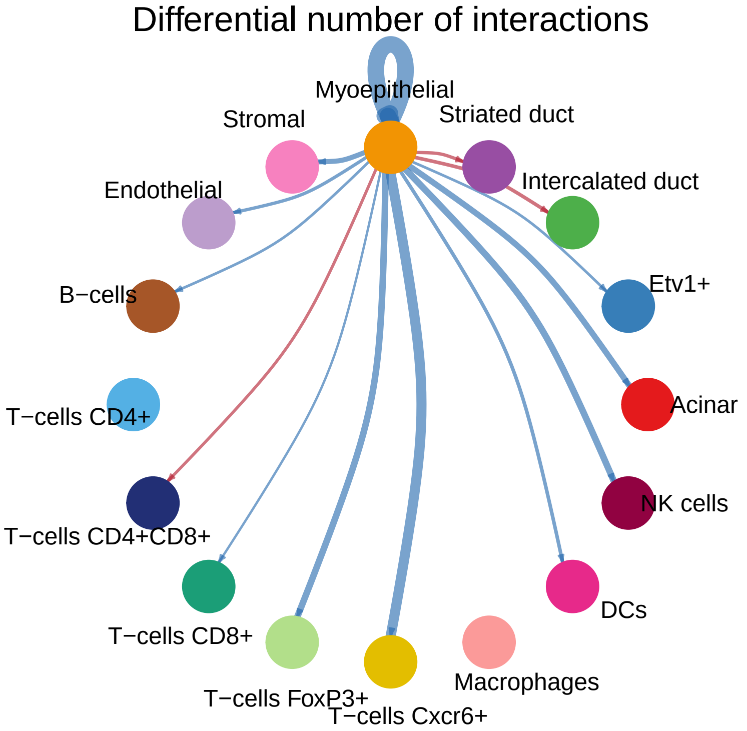

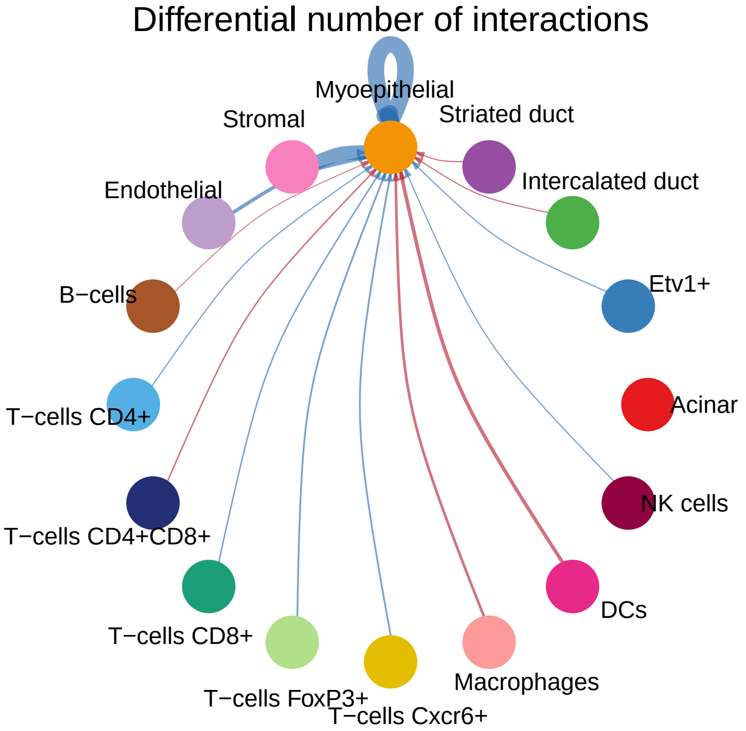

Supplemental Data S1. Cell Chat chord plots. Related to Figure 5

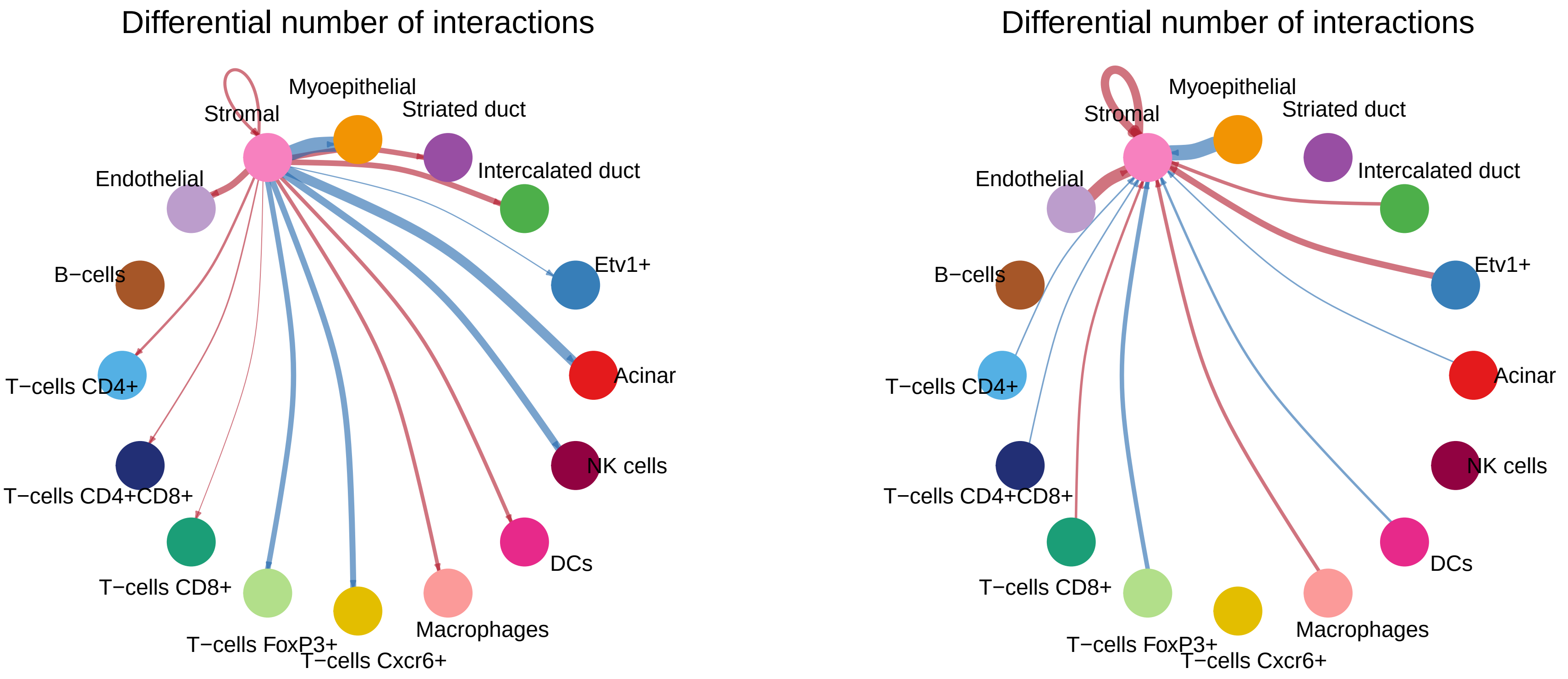

Supplemental Data S1. Cell Chat chord plots. Related to Figure 5

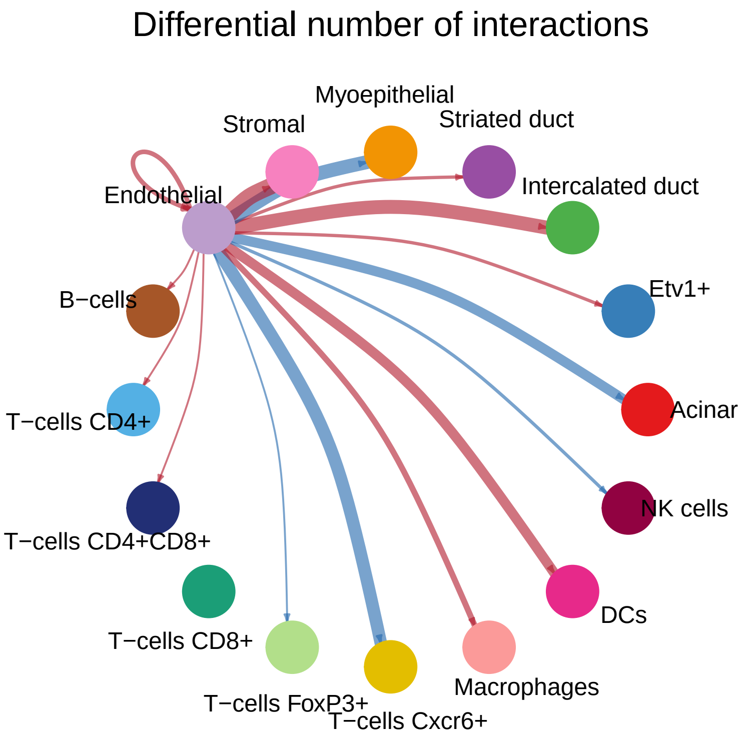

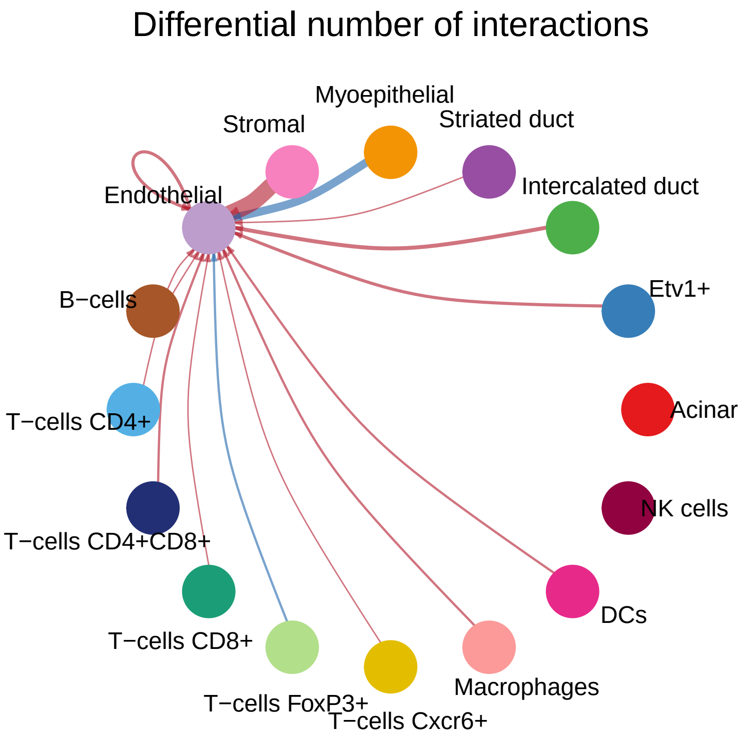

Supplemental Data S1. Cell Chat chord plots. Related to Figure 5

Differential number of interactions

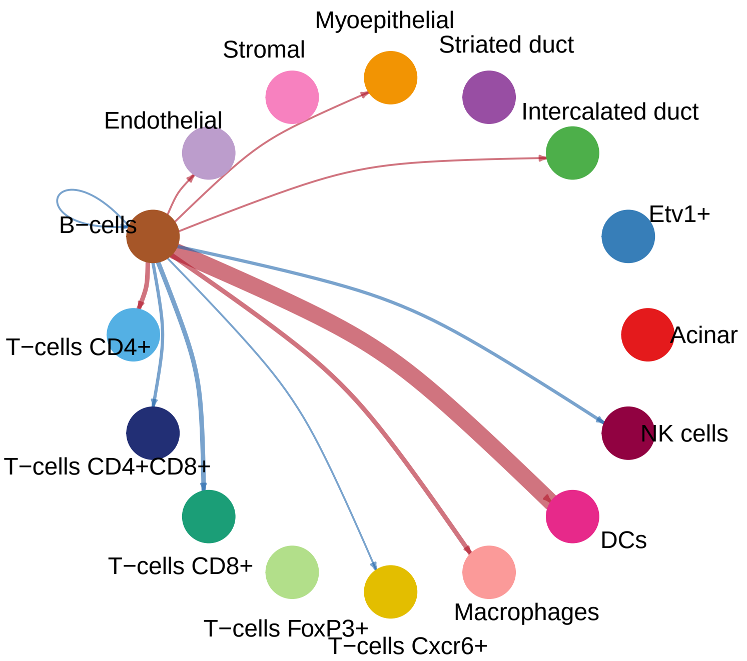

Differential number of interactions

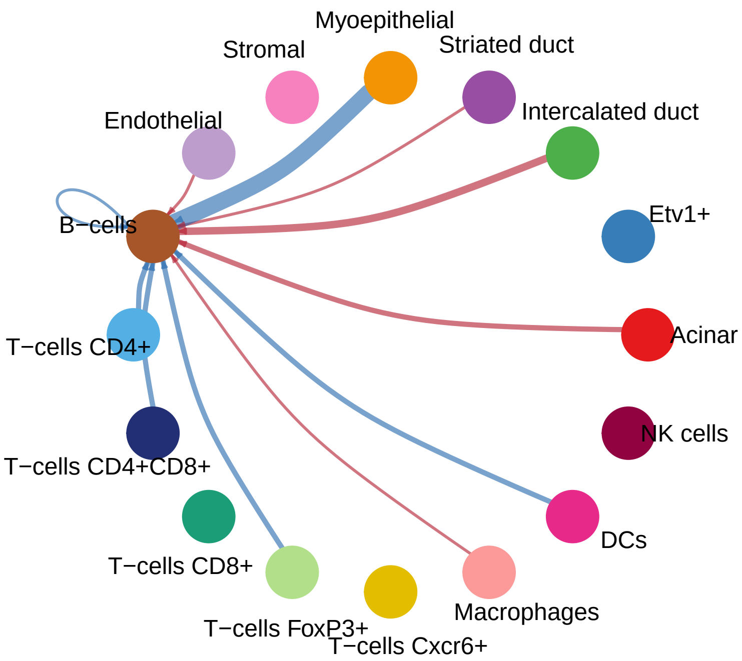

Supplemental Data S1. Cell Chat chord plots. Related to Figure 5

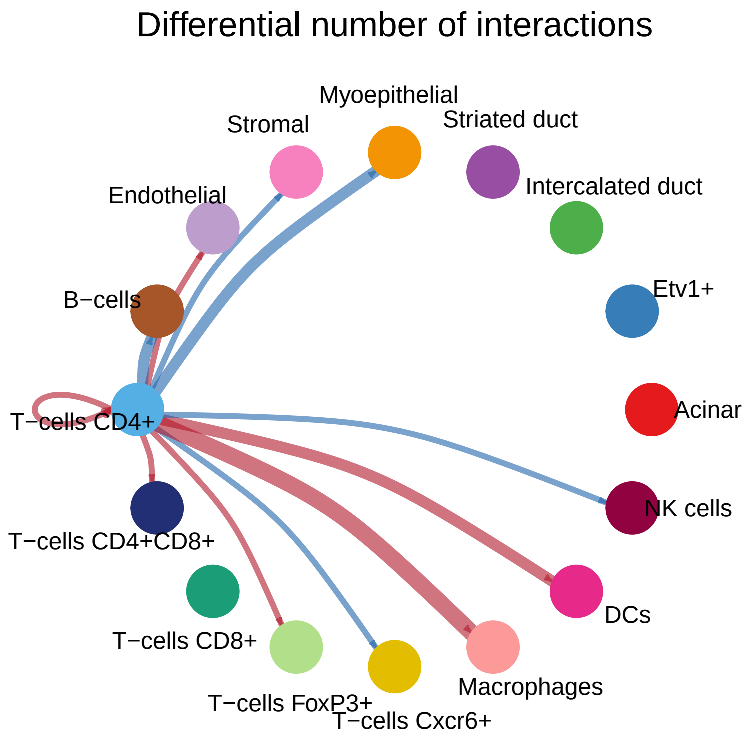

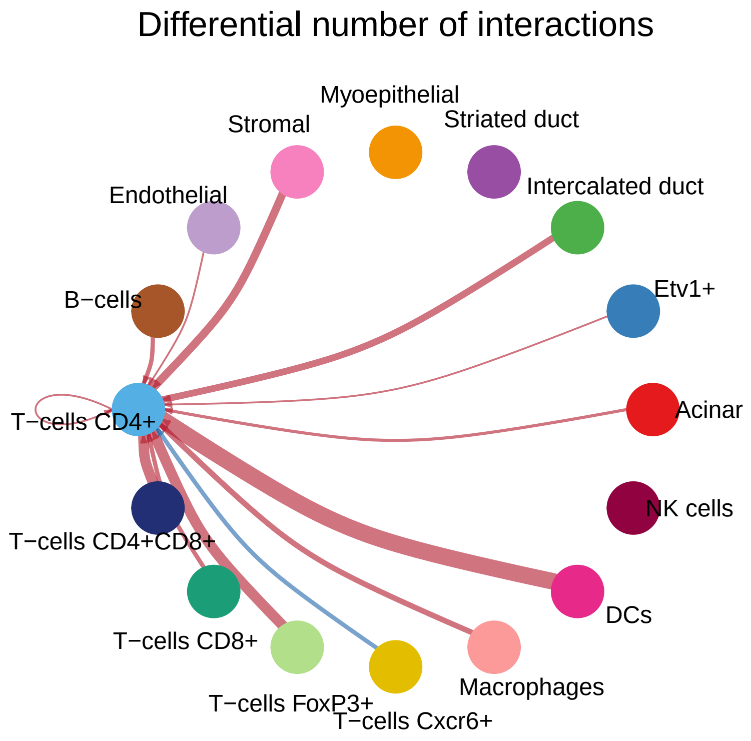

Supplemental Data S1. Cell Chat chord plots. Related to Figure 5

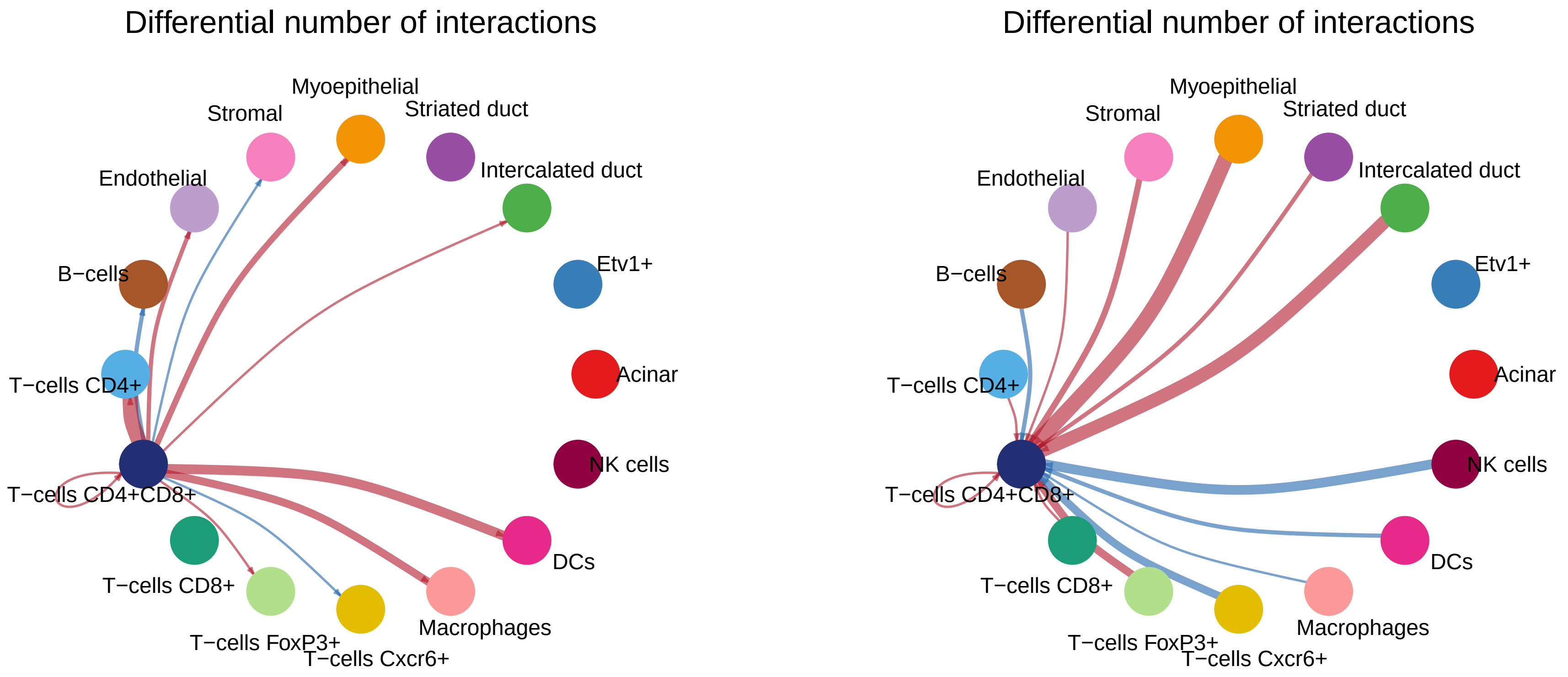

Supplemental Data S1. Cell Chat chord plots. Related to Figure 5

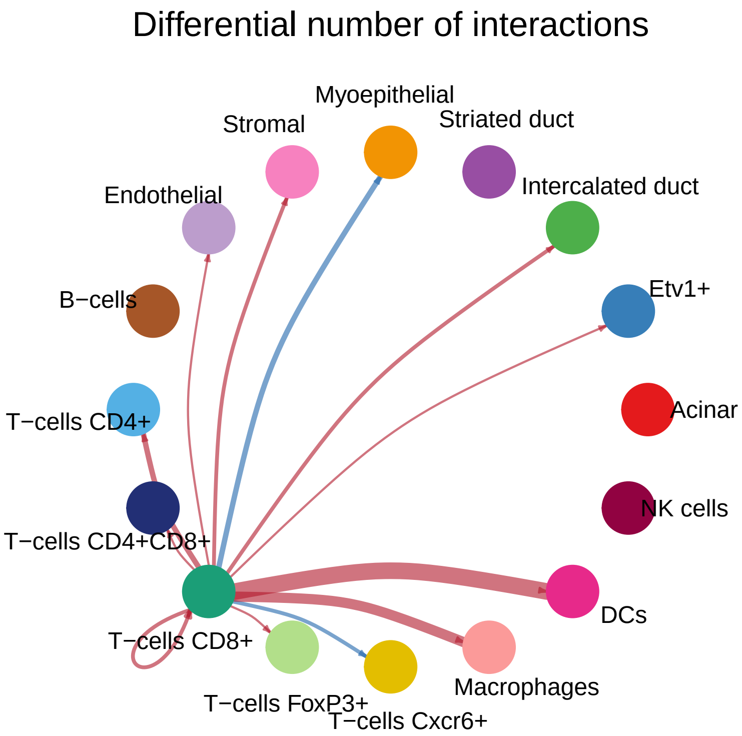

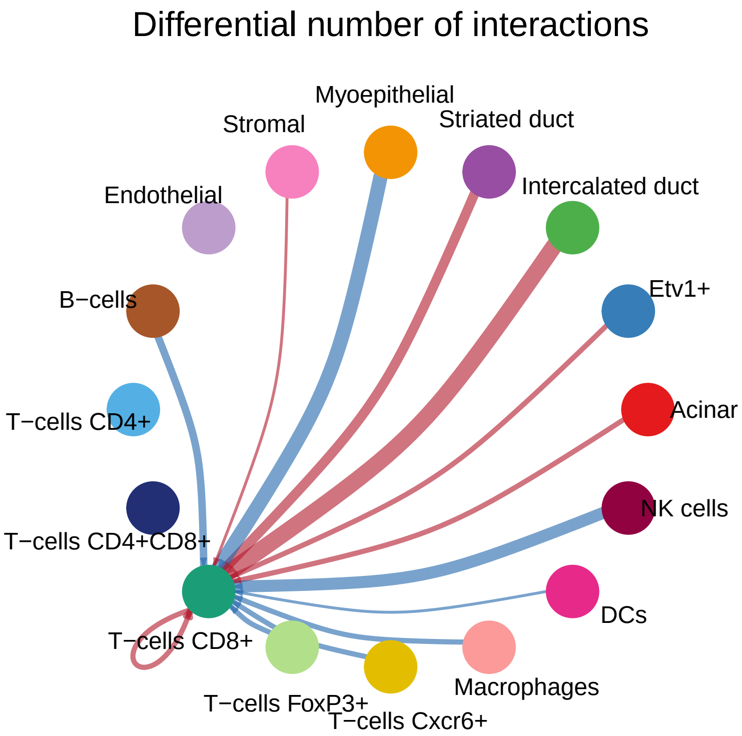

Supplemental Data S1. Cell Chat chord plots. Related to Figure 5

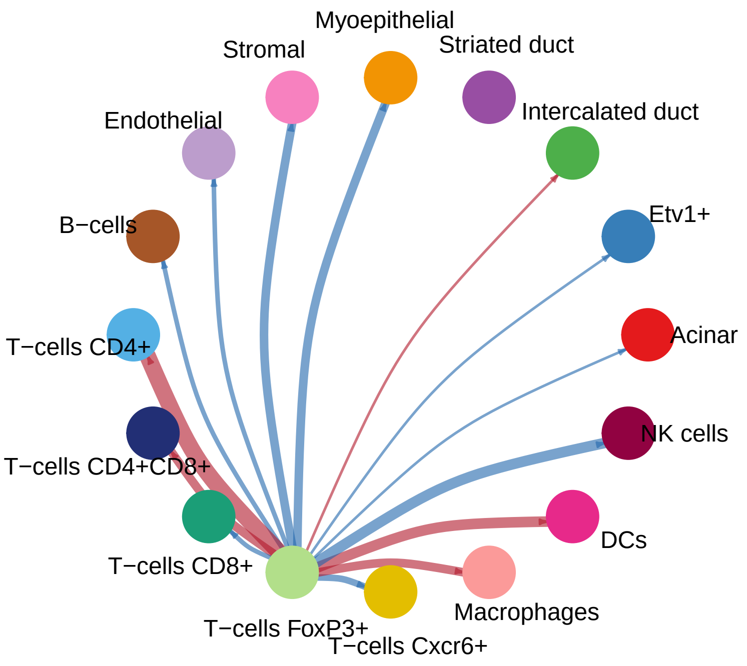

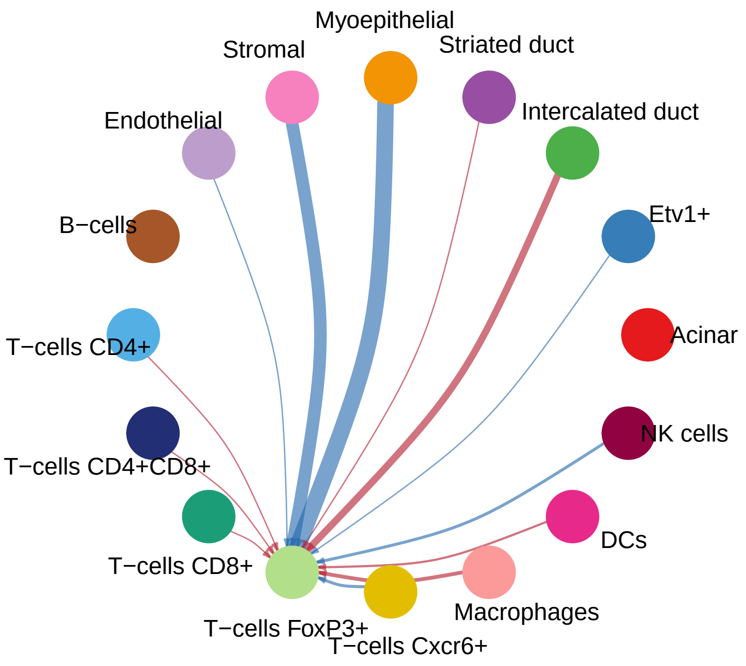

Supplemental Data S1. Cell Chat chord plots. Related to Figure 5

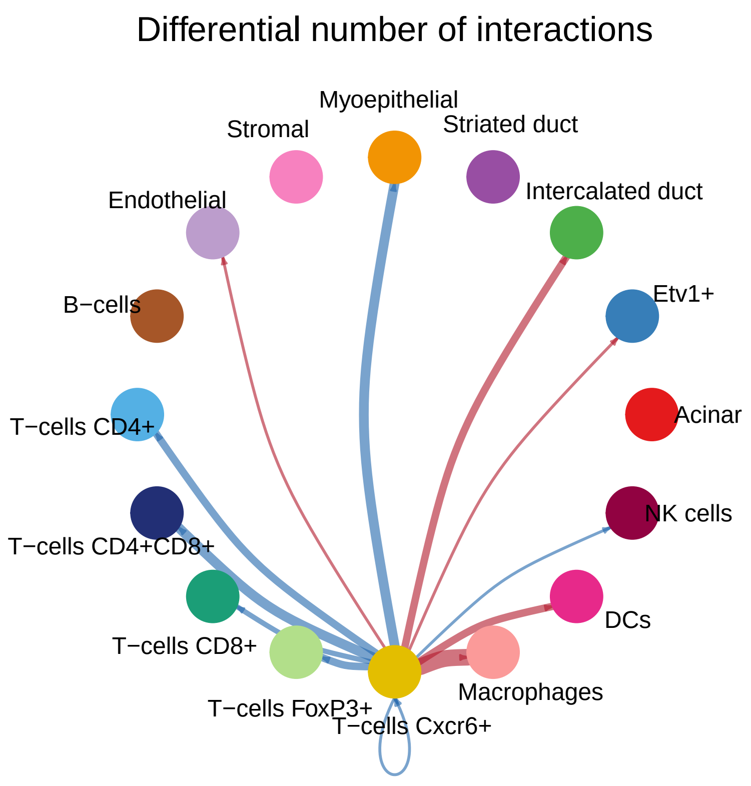

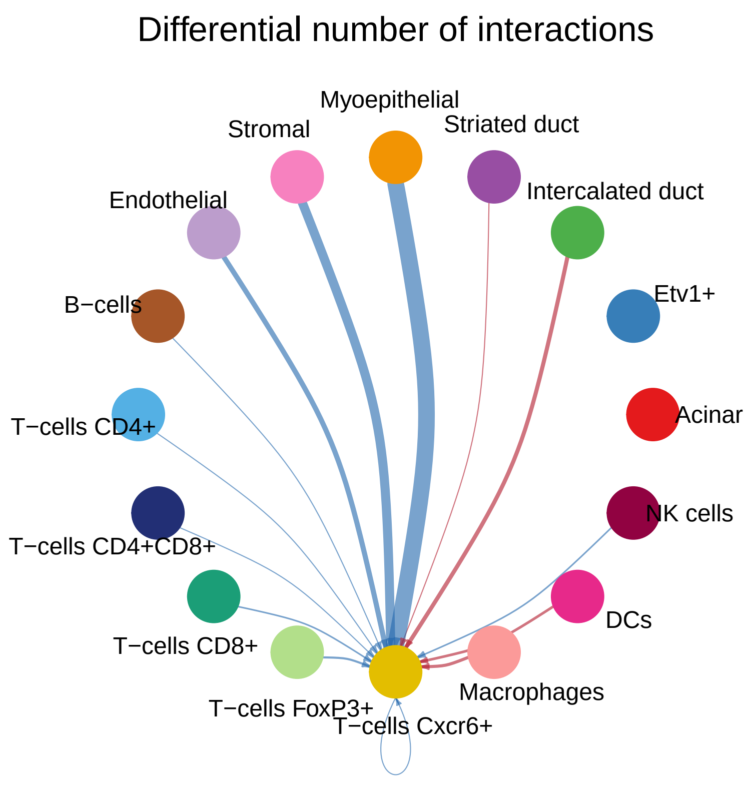

Supplemental Data S1. Cell Chat chord plots. Related to Figure 5

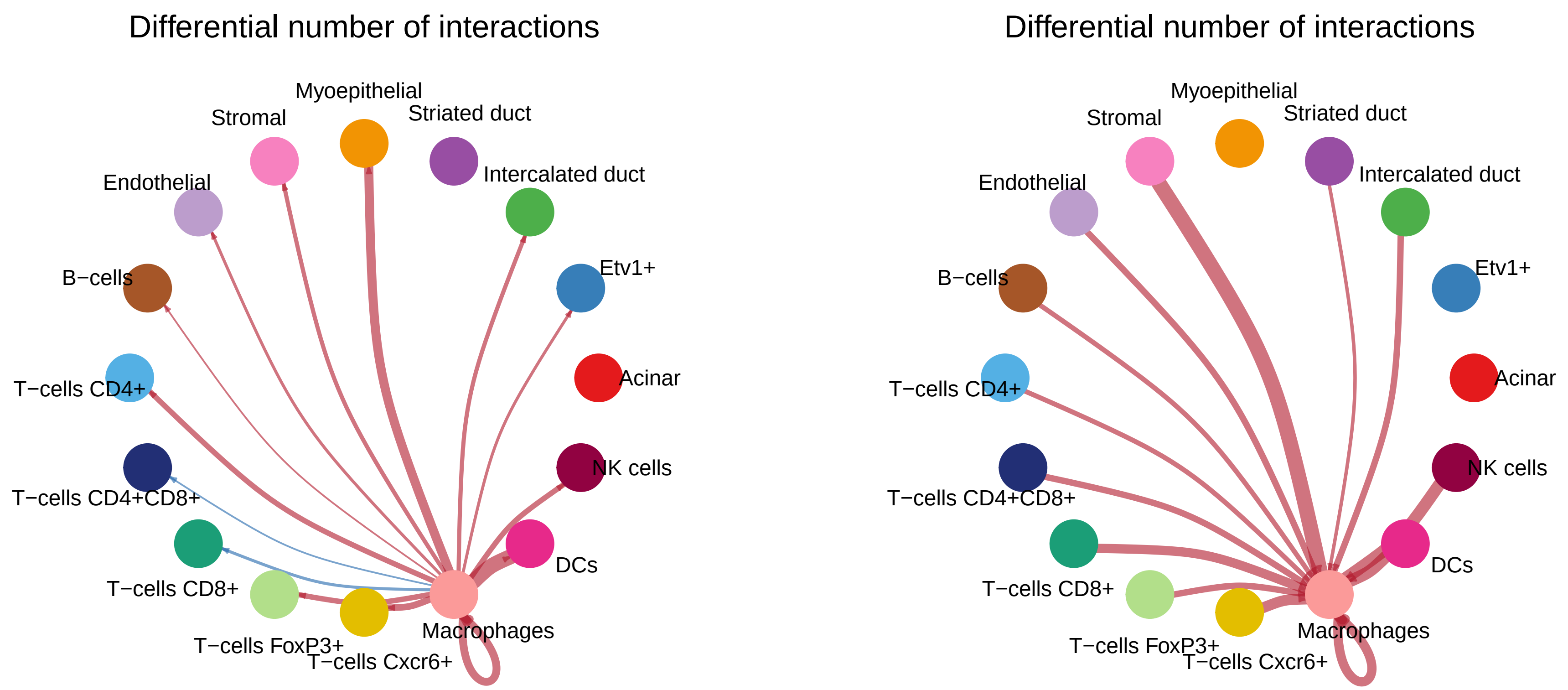

Supplemental Data S1. Cell Chat chord plots. Related to Figure 5

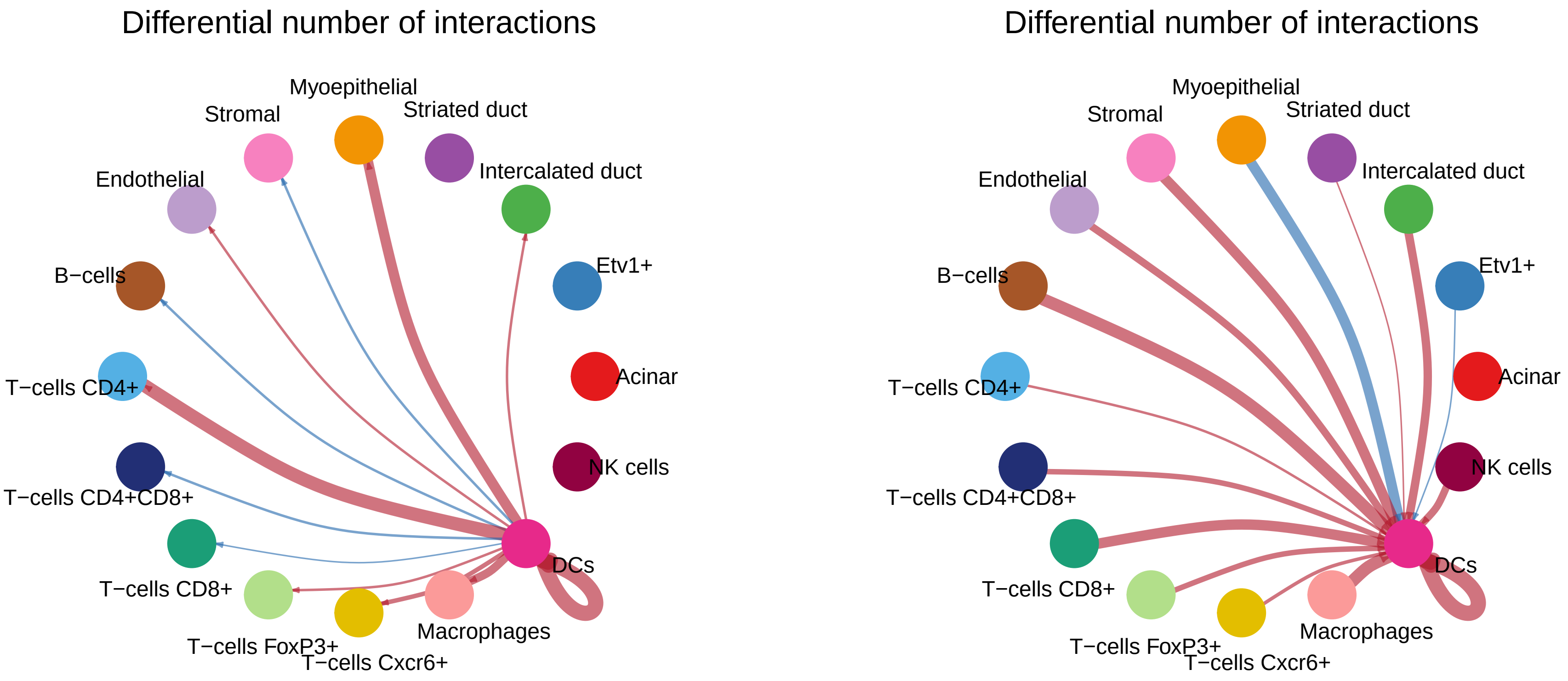

Supplemental Data S1. Cell Chat chord plots. Related to Figure 5

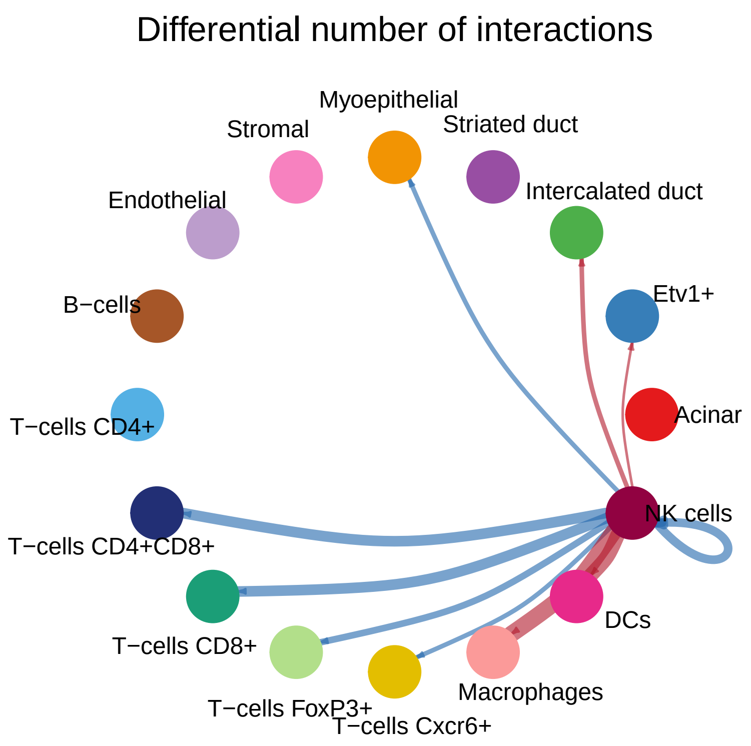

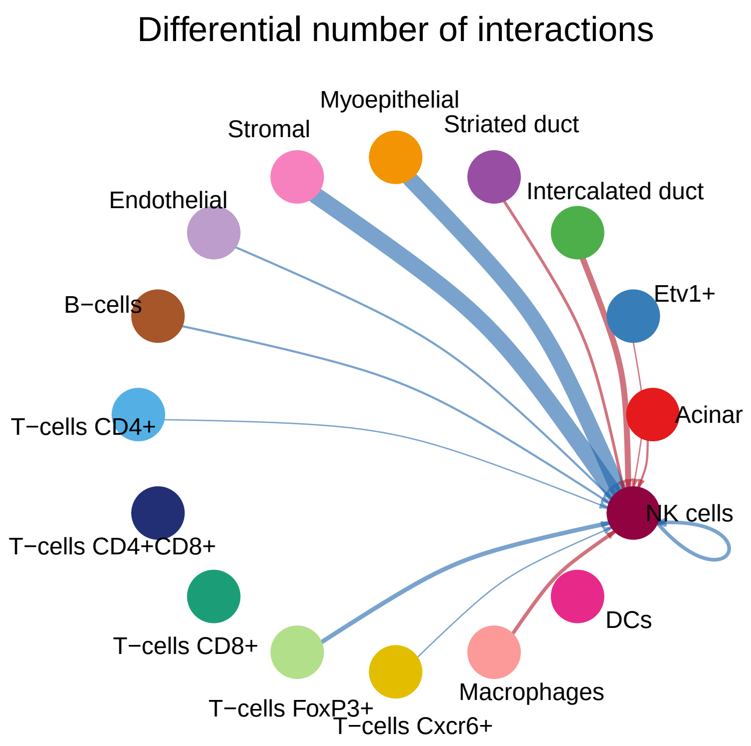

Supplement: Data S1. Cell Chat chord plots, related to Figure 5 [file mmc7.pdf]
